# Benefits and risks of antihypertensive medication in adults with different systolic blood pressure: A meta-analysis from the perspective of the number needed to treat

**DOI:** 10.3389/fcvm.2022.986502

**Published:** 2022-10-19

**Authors:** Yucheng Mao, Shiyao Ge, Sufen Qi, Qing-Bao Tian

**Affiliations:** Hebei Key Laboratory of Environment and Human Health, Department of Epidemiology and Statistics, School of Public Health, Hebei Medical University, Shijiazhuang, China

**Keywords:** blood pressure threshold, pharmacological treatment, number needed to treat, cost-effectiveness, cardiovascular endpoint

## Abstract

**Background:**

The blood pressure (BP) threshold for initial pharmacological treatment remains controversial. The number needed to treat (NNT) is a significant indicator. This study aimed to explore the benefits and risks of antihypertensive medications in participants with different systolic BPs (SBPs), and cardiovascular disease status from the perspective of the NNT.

**Methods:**

We conducted a meta-analysis of 52 randomized placebo-controlled trials. The data were extracted from published articles and pooled to calculate NNTs. The participants were divided into five groups, based on the mean SBP at entry (120–129.9, 130–139.9, 140–159.9, 160–179.9, and ≥180 mmHg). Furthermore, we stratified patients into those with and without cardiovascular disease. The primary outcomes were the major adverse cardiovascular events (MACEs), and adverse events (AEs) leading to discontinuation.

**Results:**

Antihypertensive medications were not associated with MACEs, however, it increased AEs, when the SBP was <140 mmHg. For participants with cardiovascular disease or at a high risk of heart failure and stroke, antihypertensive treatment reduced MACEs when SBP was ≥130 mmHg. Despite this, only 2–4 subjects had reduced MACEs per 100 patients receiving antihypertensive medications for 3.50 years. The number of individuals who needed to treat to avoid MACEs declined with an increased cardiovascular risk.

**Conclusion:**

Pharmacological treatment could be activated when SBP reaches 140 mmHg. For people with cardiovascular disease or at a higher risk of stroke and heart failure, 130 mmHg may be a better therapeutic threshold. It could be more cost-effective to prioritize antihypertensive medications for people with a high risk of developing cardiovascular disease.

## Introduction

Hypertension is one of the major determinants of morbidity and mortality in cardiovascular diseases (1). The global prevalence of hypertension has increased from 648 million to 1,278 million in the last 30 years (2). Since a persistent increase in the number of cases of hypertension and associated cardiovascular diseases has been significantly contributing to enormous economic ramifications, cost-effective treatment options are essential for reducing the public health burden, especially in the aged population. The timing of initiation of pharmacological treatment plays a crucial role in determining the treatment course and total healthcare expenditure (3). For decades, the blood pressure (BP) threshold of ≥140/90 mmHg is considered an indication for drug therapy in hypertensive individuals. However, in 2017, the American College of Cardiology (ACC) and the American Heart Association (AHA) guidelines recommended a BP of ≥130/80 mmHg as the cut-off for undergoing treatment in patients with high cardiovascular risks (4). Recently, an analysis from the Blood Pressure Lowering Treatment Trialists’ Collaboration (BPLTTC) suggests that pharmacological treatment should be provided to high-risk subjects with cardiovascular diseases, irrespective of their baseline BP and cardiovascular health status (5). In August 2021, the World Health Organization (WHO) recommended that 140/90 mmHg BP could still be the threshold for initiating antihypertensive medications, but with a reduction in the treatment threshold to 130–139 mmHg for individuals with existing cardiovascular diseases (6).

Meta-analysis is an effective method to support developing and modifying clinical guidelines (7, 8). However, most meta-analyses have typically reported relative indicators, such as the relative risk and odds ratio. Relative indicators are flawed in their abilities to reflect outcomes in populations who have not received any treatments of interest. Thus, these indicators are not overly effective in evaluating healthcare investments and expenses. The “control event rate (CER)” measurement is a very important method of estimation as it avoids overestimation. For example, if the ratio of the target outcome in the treatment group is 1 event per 1,000 events while that in the control group is 2 events per 1,000 events, the result can be reported as a twofold decrease when using relative indicators. However, the absolute difference in the event rate is only 1/1,000, which gives a different insight. The number of patients needed to treat, defined as the inverse of the absolute risk difference, reflects the CER (9). The number needed to treat (NNT) refers to the number of individuals who should be treated with a given therapy when avoiding one adverse event (AE), providing more intuitive information in interpreting research data. It shows the efficacy and safety parameters as well as the total treatment cost, and many top-tier medical journals suggest that NNT should be used in drug evaluation (10).

Number needed to treat is rich in applications for a wide range of chronic diseases (e.g., hyperlipidemia, prostate cancer, depression, etc.) (11–13). A previous meta-analysis on the antihypertensive medication in people with different BPs has reported the NNT, but the risk related to treatment and differences between participants with and without previous cardiovascular diseases has not been investigated in detail (14). Other existing studies on the NNT primarily evaluate the BP lowering efficiency among different classes of antihypertensive drugs, instead of different treatment thresholds (15–18).

Therefore, we aimed to simultaneously explore the benefits and risks of antihypertensive medications, in the context of NNT, in people with different baseline values of systolic BP (SBP) and cardiovascular disease.

## Materials and methods

### Search strategy and selection criteria

This systematic review was performed according to the Preferred Reporting Items for Systematic Reviews and Meta-Analyses (PRISMA) guidelines. PubMed, Web of Science, Science Direct, The Cochrane Library, and Clinical Trials databases were searched for articles published in English or Chinese up to “November 2021.” We used a combination of Medical Subject Headings (*MeSH)* terms, in addition to free terms to improve the overall recall ratio. The reference lists of included large-scale meta-analyses were screened to identify any additional studies (Supplementary Method 1).

Parallel-group randomized controlled trials were eligible if they (1) randomized assignments to placebo versus five classical antihypertensive drugs; (2) recruited adult participants to the study; (3) had a follow-up duration of ≥1 year; and (4) reported one of the outcomes of interest. Notably, trials that only included participants with acute cardiovascular diseases were excluded. Two reviewers (YM and SG) worked independently to screen studies that fulfilled the selection criteria. Selection criteria are described in Supplementary Method 2.

### Data extraction and quality assessment

All data were extracted independently by a single investigator (YM) and cross-verified by the investigator (SG) using the same data extraction Excel form. If these two investigators met with controversies, a third reviewer (SQ) was consulted to make a final decision. The primary endpoints were the major adverse cardiovascular events (MACE), defined as death related to cardiovascular diseases (CVDs), myocardial infarction (MI), stroke, and withdrawal due to AEs. Reported AEs mainly included hypotension, peripheral edema, cough, renal dysfunction, hyperkalemia, dialysis, dizziness, angioedema, uncontrolled hypertension, syncope, headache, diarrhea, nausea, vomiting, bradycardia, shortness of breath, fatigue, and cold extremities. The secondary endpoints were all-cause death (ACD), CVD, MI, heart failure (HF), and stroke. The following information was extracted from each eligible study: trial name, year of publication, number of participants in total and in each arm, mean follow-up duration, mean age of participants, percentage of females, mean body mass index (BMI), mean BP at entry, change in BP after treatment, the difference in BP reduction between the treatment and control groups, and the number of subjects who did and did not fulfill the primary and secondary endpoints in each arm. The study quality was assessed independently in duplicates by two investigators (YM and SG) using the Cochrane risk of bias assessment tool.

### Statistical analysis

We analyzed data based on the intention-to-treat principle. SBP is associated with cardiovascular events among people of all ages and is particularly important among older age groups. DBP, however, appears to be a relatively weaker risk factor for cardiovascular events in older populations. The mean age of participants in the current study was 61.52 years which we feel justifies the prioritization of SBP (19). In general, the SBP of ≤120 mmHg is considered normal, and 120–139 mmHg is the normal high range. While 140–159 mmHg is referred to as the criteria for the diagnosis of grade 1, 160–179 mmHg for grade 2, and ≥180 mmHg for grade 3 hypertensive disorders. However, both the ACC and AHA have recommended reducing the threshold for treatment initiation to ≥130/80 mmHg. To explore the benefits and risks of antihypertensive medications in participants with different SBP values, we divided SBP into five ranges for risk classification: 120–129.9, 130–139.9, 140–159.9, 160–179.9, and ≥180 mmHg. The NNT can also be described as the number needed to be treated for an additional beneficial (NNTB) outcome and the number needed to be treated for an additional harmful (NNTH) outcome (20). The computation of the NNT was based on the cumulative incidences of the outcome results when the target events were time-related (e.g., mortality) (21, 22). However, the proportion of patients with the outcome showed a minor effect on the final results if the observation duration was set shorter or the study had fewer lost-to-follow-up participants (22). The included trials were relatively short with a maximum follow-up duration of 5.8 years and few participants (2.67%) were lost-to-follow-up. Thus, the NNT was calculated using the formula: NNT = 1 ÷ (Pc − Pi), where Pc and Pi indicated the proportion of patients with target events in the control group and the treatment group, respectively (20). All NNT values were rounded up to the nearest whole number (23). We calculated NNT together with its 95% confidence interval (CI). If the 95% CI reached “infinity,” the result was considered non-significant (23).

The NNT is preferably computed by the pooled relative effects (e.g., relative risks) in a meta-analysis (24). The formula used to convert the relative risk into the NNT is as follows, NNT = 1 ÷ [(1 − relative risk) × CER] (20). The corresponding CIs for the NNT can be calculated by the upper and lower confidence limits for the pooled relative risks and the value of pooled CER (20). The fixed effects model or the random effects model was applied according to the degree of heterogeneity, such as *I*^2^ ≤ 50% for fixed effects model, and *I*^2^ > 50% for random effects model.

The NNT is highly related to the length of the observation period (21). Thus, the NNT should be standardized when making comparisons between studies involving different observation periods (9). Of note, the NNT with an observation period of T years can be converted into an approximate equivalent with a standard duration of S years using the formula: NNT: S = NNT: T × T/S, where NNT: T is the observed NNT in each trial, T/S is the ratio of the observed follow-up duration to the mean follow-up duration among the included trials, and NNT: S is the NNT after standardization (25).

Accordingly, we segregated the participants based on their reported cardiovascular disease status, which included HF, MI, atrial fibrillation, stroke, coronary artery disease (CAD), and coronary artery bypass grafting (CABG). Trials with mixed populations were classified as cardiovascular disease trials if ≥50% of the participants had a history of cardiovascular disease. We conducted a sensitivity analysis to assess the robustness of the findings and a meta-regression analysis to test the influence of baseline characteristics on the MACE. Publication bias was evaluated using the standard funnel plots. Egger’s test was used to determine the magnitude and statistical significance of the relationship between the observed effect sizes and the study sizes. The significance level was set at *P* < 0.05. All statistical analyses were carried out using Stata version 16.0 and R version 4.1.1.

## Results

### Literature search and baseline characteristics

We retrieved 11,590 articles and 1,744 clinical trials from relevant databases. Out of which, 54 studies were eligible for this meta-analysis (26–79). However, two studies had no information of the baseline systolic blood pressure, finally, 52 studies (including 58 comparison groups), corresponding to 213,342 participants, were analyzed in the meta-analysis (26, 29–79). The retrieval process is presented in Supplementary Figure 1. Studies were divided between the primary preventive [34 (65.38%) of 52] and cardiovascular disease secondary prevention [18 (34.62%) of 52] groups. Females occupied 39.77% of the participants. The average participant age was 61.52 years. The mean SBP and mean diastolic blood pressure (DBP) at entry were respectively 152.08 and 87.13 mmHg. Mean BP in those with previous cardiovascular disease occurrences (138.47/81.36 mmHg) was much lower than in those without (159.24/90.25 mmHg). The follow-up duration ranged from 1 to 5.8 years, with a mean duration of 3.55 years (Table 1). The risk-of-bias varied across studies, and the overall quality of enrolled studies was satisfactory (Supplementary Figure 2). Baseline characteristics of all studies are shown in Supplementary Table 1.

**TABLE 1:** Baseline characteristics of participants by cardiovascular disease status and systolic blood pressure.

	Total participants (*n* = 213,342)	Participants without previous cardiovascular diseases (*n* = 115,030)	Participants with previous cardiovascular diseases (*n* = 98,312)
**Sex**			
Women (%)	39.77	43.76	32.39
Age (years)	61.52 (9.16)	60.78 (10.94)	62.92 (3.96)
Systolic blood pressure (mmHg)	152.08 (18.60)	159.24 (17.89)	138.47 (10.82)
Diastolic blood pressure (mmHg)	87.13 (9.61)	90.25 (9.74)	81.36 (6.21)
**Categories of systolic blood pressure (mmHg)**			
120–129.9	7,774 (3.64%)		7,774 (7.91%)
130–139.9	74,621 (34.98%)	21,948 (19.08%)	52,673 (53.58%)
140–159.9	70,316 (32.96%)	32,903 (28.60%)	37,413 (38.06%)
160–179.9	50,528 (23.68%)	50,076 (43.53%)	452 (0.46%)
≥180	10,103 (4.74%)	10,103 (8.78%)	
BMI	28.00 (1.88)	27.77 (2.12)	28.56 (0.97)
Follow-up (years)	3.55 (2.60–4.50)	3.37 (2.83–4.40)	3.9 (2.55–4.67)

Data are *n* (%), mean (SD), or median (Q1-Q3), unless otherwise specified. BMI, body mass index; follow-up, follow-up duration in years.

### Primary outcomes

**FIGURE 1:**
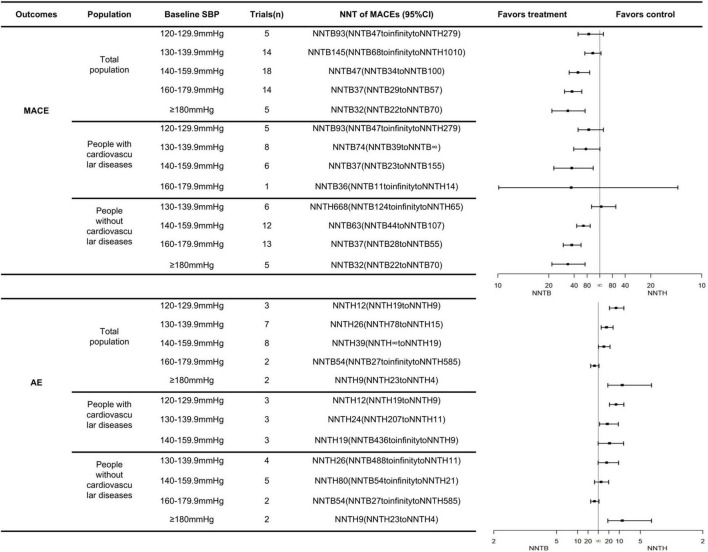
The NNTs for the primary outcomes across different SBP groups and cardiovascular diseases status.

The number needed to be treated for an additional beneficial outcome and NNTH analyses demonstrated the benefits and risks of treatments, respectively. A more favorable treatment profile might be associated with a lower NNTB and a higher NNTH value. For participants with an SBP <140 mmHg, antihypertensive medications were not associated with any effects on the MACE but might have an association with an increased risk of AEs leading to withdrawal. At an SBP range of 140–159.9 mmHg, antihypertensive medication was recommended, and the expected benefits were close to the possible risks (NNTB 47, 95% CI: NNTB 34 to NNTB 100 vs. NNTH 39, 95% CI: NNTH ∞ to NNTH 19). For participants with an SBP range of 160–179.9 mmHg, BP lowering medications had positive effects on the MACEs (NNTB 37, 95% CI: NNTB 29 to NNTB 57), which meant that 37 patients needed to be treated for 3.50 years to prevent one patient from experiencing the MACEs. At this SBP level, antihypertensive treatment did not increase the risks of AEs (the 95% CI of the NNT included infinity). The positive effect of the antihypertensive treatment on the MACEs in the SBP ≥180 mmHg group was close to that of the group having an SBP range of 160–179.9 mmHg (NNTB 32, 95% CI: NNTB 22 to NNTB 70 vs. NNTB 37, 95% CI: NNTB 29 to NNTB 57). However, antihypertensive treatment might significantly increase risks of AEs, if the initial treatment is provided until the SBP reaches ≥180 mmHg (NNTH 9, 95% CI: NNTH 23 to NNTH 4) (Figure 1).

**FIGURE 2:**
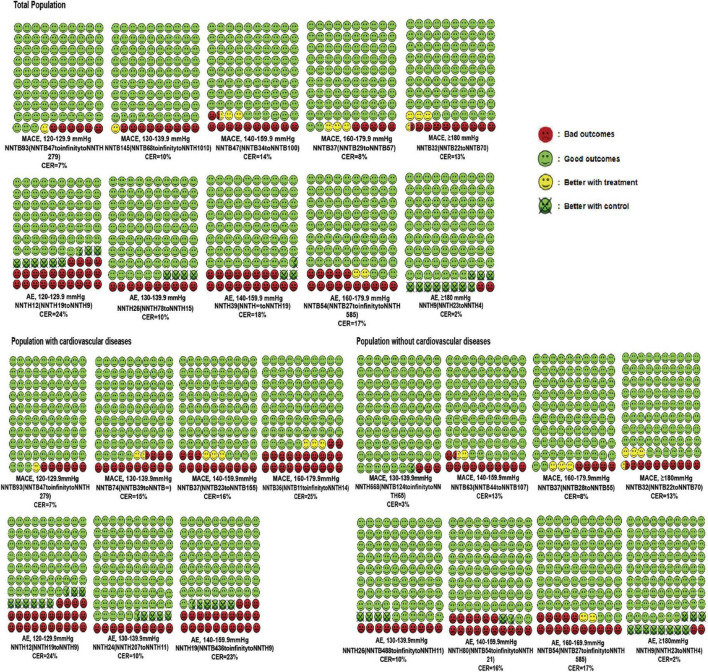
Cates plot of the NNTs for primary outcomes across different SBP levels and cardiovascular diseases status. Each region is the value of NNT of MACEs or AEs in one SBP level and includes 100 faces corresponding to the patients treated with antihypertensive medication. Green faces mean patients not occurring MACEs or AEs with the treatment. Red faces indicate that patients presenting MACEs or AEs with the treatment. Yellow faces represent patients that would not have MACEs or AEs if they would be treated with the treatment. Crossed green faces present patients not fulfilled MACEs or AEs with a control group. Mean follow-up duration was 3.50 years.

Likewise, the treatment threshold was set at an SBP of 140 mmHg among patients undergoing primary prevention with an NNTB value of 63 (95% CI: NNTB 44 to NNTB 107). In participants with previous cardiovascular disease, the antihypertensive treatment reduced the MACE when SBP reached a range of 130–139.9 mmHg with an NNTB value of 74 (95% CI: NNTB 39 to NNTB ∞). It was worth noting that the number of people who received direct cardiovascular benefits from using antihypertensive medications was not very significant. The number of “yellow faces” in Figure 2 vividly represents the number of patients who could avoid the MACEs if 100 patients were treated with the antihypertensive medication (detailed interpretation is supplied in the figure legends). Figure 2 suggests that only 2–4 subjects had reduced the MACEs per 100 subjects receiving BP-lowering drugs for 3.50 years. Details of the calculation are shown in Supplementary Table 2.

### Secondary outcomes

**FIGURE 3:**
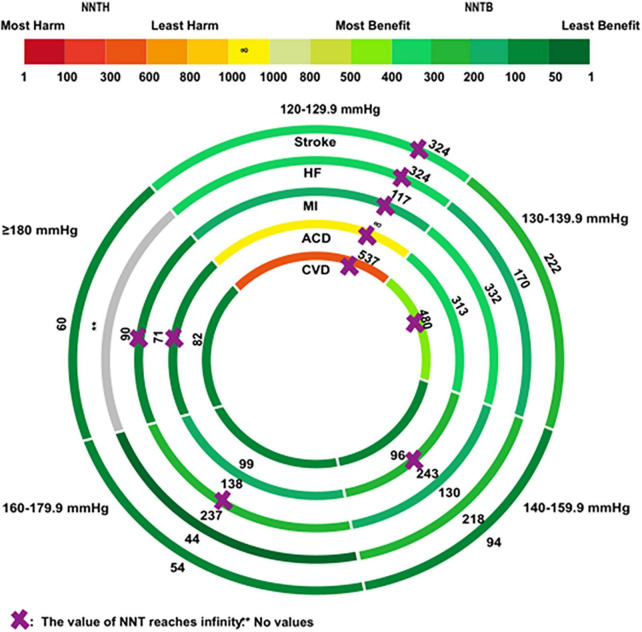
The rank-heat plot of the NNT values for secondary outcomes across five baseline SBP levels. Circles from outside in refer to first, stroke; second, heart failure (HF); third, myocardial infarction (MI); fourth, all-cause death (ACD); fifth, cardiovascular death (CVD). Sectors with a “purple cross” mean that the values of NNT reach infinity. The sector with a “two asterisks” means that there is no values in the group. Each section is colored according to the NNT value of the corresponding SBP and outcome. The scale consists of the transformation of three colors red (NNTH = 1), yellow (NNTB/NNTH = ∞), and green (NNTB = 1). The redder the color of the section, the smaller the NNTH is; the greener the color of the section, the smaller the NNTB is. Each section also includes the NNT value corresponding to the specific baseline SBP and outcome. Mean follow-up duration is 3.50 years.

Figure 3 visualizes the NNT of secondary outcomes in the total population. Antihypertensive medications had no positive effects on the five secondary outcomes when SBPs were at a range of 120–129.9 mmHg. Antihypertensive medications reduced the risk of CVD when SBP was 140 mmHg, with an NNTB value of 96 (95% CI: NNTB 56 to NNTB 1052), an NNTB of 99 (95% CI: NNTB 66 to NNTB 254) and an NNTB of 82 (95% CI: NNTB 49 to NNTB 434), respectively, in the groups with an SBP of 140–159.9 mmHg, 160–179.9 mmHg, and ≥180 mmHg. BP-lowering was not linearly associated with ACD. Antihypertensive medications exhibited a positive effect on ACD when at SBP ranges of 130–139.9 mmHg, and 160–179.9 mmHg, but no such effects were reported when SBP ranges were 140–159.9 mmHg, and ≥180 mmHg. Either very high or low BP could impose an unfavorable cardiac condition, leading to MI, where BP-lowering treatment was beneficial only in the patient group having an SBP range of 130–159.9 mmHg, but not in the group with <130 mmHg or >160 mmHg. Abnormal BP changes have been well-characterized for the development of stroke and HF. Antihypertensive treatment showed positive effects on stroke, and HF symptoms when the SBP was >130 mmHg, with an NNTB ranging from 222 to 54 and an NNTB ranging from 218 to 44, respectively. The detailed calculations are supplied in Supplementary Table 3.

### Relationship between the number needed to treat and the control event rate of major adverse cardiovascular events

**FIGURE 4:**
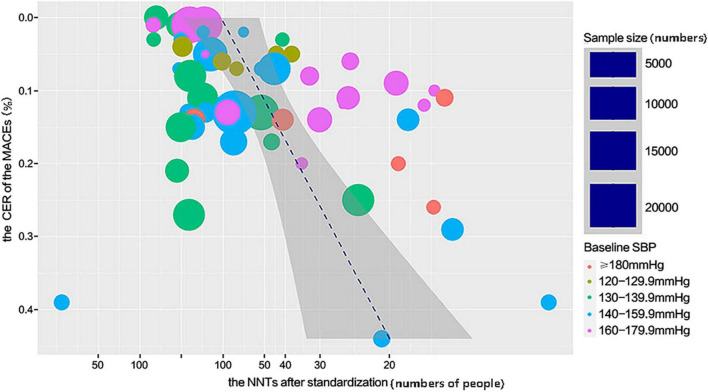
The relationship between the NNT of the MACEs and the CER of the MACEs.

The relationship between the NNT and CER of MACEs follows the model shown in Figure 4. The number of individuals who needed to treat to avoid MACEs significantly declined with an increased risk of MACEs. Under the same condition, the lower NNT could be beneficial for lowering the treatment cost to achieve the expected outcome. According to this model, it could be more cost-effective to prioritize antihypertensive medications for individuals with a high risk of cardiovascular events. Calculated data are provided in Supplementary Table 4.

### Sensitivity analysis and publication-bias

Our main findings were precise and reproducible in the sensitivity analysis (Supplementary Figure 3). The meta-regression analysis revealed that antihypertensive drug effects on MACEs did not differ among baseline characteristics except for BMI (Supplementary Table 5). We could not find any evidence of publication-bias (Supplementary Figure 4).

## Discussion

Our data suggest that an SBP of 140 mmHg might be the threshold for initiating the primary pharmacological treatment, however, 130 mmHg might be a better therapeutic threshold in patients with a history of cardiovascular disease or who are at a higher risk of stroke and HF. Despite these facts, the number of subjects who received cardiovascular benefits directly from antihypertensive medications was not significantly large. It could be more cost-effective to prioritize antihypertensive medication for individuals with a high risk of cardiovascular events. Notably, the value for the NNTB of MACEs (NNTB 47) did not differ significantly with respect to the value for the NNTH of AEs (NNTH 39) among participants with SBPs ranging 140–159.9 mmHg, indicating that the benefits and risks of antihypertensive treatments were similar for subjects having SBPs within that range. However, the benefits were only confined to reducing the occurrence of MACEs, while the risks were concentrated on the AEs leading to a withdrawal in our study. Other benefits (e.g., cost saving), and risks (e.g., AEs that did not result in withdrawal) were not considered in this analysis. It should also be pointed out that only one trial was included in the analysis where cardiovascular disease patients had baseline SBP of 160–179.9 mmHg. However, the results might not be robust enough to draw any solid conclusion, given the limited number of studies included in the analysis. More trials focusing particularly on this SBP level should be analyzed in the future toward a statistically significant conclusion. Besides, the 95% CI for the treatment effect on CVD was large among subjects within the SBP range of 140–159.9 mmHg. By contrast with the relative risk, the NNT reflects the absolute effect difference between the two groups. The large CI might be associated with the upper and lower limits of the absolute difference between the event rate in the control group (CER) and that in the treatment group (EER), according to the NNT calculation formula. If the absolute difference (CER-EER) is very small, the NNT (the reciprocal of the “CER-EER”) tends to be very large. For participants with an SBP of 140–159.9 mmHg, the upper limit 95% CI of the difference between CER and EER was only 0.001 (0.004 in SBP of 160–179.9 mmHg and 0.003 in SBP of ≥180 mmHg), resulting in a very large upper limit 95% CI of the NNT (calculated based on the data presented in Supplementary Table 2). Moreover, in our study, discrepancies in antihypertensive agents were evident. Angiotensin-converting enzyme inhibitor (ACEI) was the most common therapy, followed by angiotensin receptor blocker (ARB). Combined administration of the diuretic and β-blocker was the most common double-drug combination therapy. Different therapies might have different effects on the overall results.

Our results were consistent with a meta-analysis, illustrating that the primary preventive antihypertensive treatment has no cardiovascular benefits but rather increases the risk of AEs for patients with an SBP range of 130–140 mmHg (8). Similarly, the 2021 WHO guidelines suggested that the timely initiation of pharmacological antihypertensive treatment in individuals diagnosed with hypertension and an SBP >140 mmHg or DBP >90 mmHg is crucial, but the threshold can be reduced to 130 mmHg for subjects with a history of cardiovascular diseases (6). Compared with the 2021 WHO guidelines, the European Society of Hypertension (ESH)/European Society of Cardiology (ESC) guidelines from 2018 and the International Society of Hypertension (ISH) guidelines from 2020 took more account of the effect of the cardiovascular risk levels, recommending that people with a SBP of 140 mmHg or DBP of 90 mmHg and a high cardiovascular disease risk are advised to take antihypertensive medications (80). Nevertheless, the results differ from an individual meta-analysis study conducted by the BPLTTC, emphasizing that each 5 mmHg decrease in SBP could be associated with an approximately 10% of the decrease in MACEs, even for participants with normal SBP or without cardiovascular disease history (5). This individual meta-analysis not only included placebo-controlled trials but also active-controlled trials (5). However, a comprehensive search was not performed in this study, and only 21 placebo-controlled trials were included. Moreover, BPLTTC refers to a different definition of MACEs, potentially leading to different results.

Notably, cardiovascular benefits may vary among populations with different levels of cardiovascular risks. The CAMELOT (with an annual cardiovascular event rate of 11.6%) study enrolled participants had a history of CAD but no hypertension, indicating that antihypertensive treatment could reduce the risk of adverse cardiovascular events (75). The Heart Outcomes Prevention Evaluation-3 (HOPE-3) study targeted subjects with intermediate cardiovascular risks independent of hypertension. In HOPE-3 (with annual cardiovascular event rate of only 0.8%), BP-lowering revealed no benefit on adverse cardiovascular events (81). A Cochrane review supports for people with relatively low risk of experiencing adverse cardiovascular events, antihypertensive medication did not reduce the occurrence of ACD, CVD, and stroke (82). Another research also supports for people with mild hypertension (140–159/90–99 mmHg) and low cardiovascular risks, lifestyle modification instead of antihypertensive drug therapy may offer more benefits and better treatment outcomes (83). Thus, whether the results from participants with a high risk of cardiovascular events can be extrapolated to other populations with an intermediate or low cardiovascular risk may be weighed more carefully.

The major strength of this study was that the study considered the effect of the CER. To the best of our knowledge, this meta-analysis was the first to simultaneously assess the benefits and risks of antihypertensive treatments in terms of five different baseline SBP groups and cardiovascular disease status from the perspective of NNT. Populations with and without diagnostically confirmed cardiovascular disease may have different cardiovascular physiological states. Extrapolating results from patients with established cardiovascular disease to the population without that is likely to introduce flaws in the analysis. Thus, analyzing these two populations separately should be seriously considered to obtain reliable and reproducible results. Moreover, we demonstrated the standardization of the NNT parameter when comparing trials with different follow-up durations, further ensuring the objectivity and accuracy of our data. Finally, all included studies were randomized placebo-controlled trials, which clearly reflected the actual efficiency of respective therapies and secured the results against the impact of other drug interferences.

Our study also has several limitations that should be carefully noted. First, the meta-analysis was based on the study-level analysis. Studies were only included based on the average SBP values, which meant that several individuals outside the reference SBP ranges might have been included in this meta-analysis. The same problem was encountered in categorizing the cardiovascular disease status. Thus, trials with mixed populations were classified as secondary prevention trials if ≥50% of the participants previously had cardiovascular disease. Subjects with previous cardiovascular disease might have mistakenly demarcated into the primary prevention group or vice versa. Second, the NNT should be calculated with the consideration of time. However, due to the lack of individual follow-up duration data, we determined the mean follow-up duration of these trials and included them in our analysis. Moreover, the mean duration of 3.55 years of trials included in this study was relatively short, and several endpoints might not be observed during the observation. Finally, the number of individuals who had a reduction in the MACE due to the use of antihypertensive medication was not very significant. In other words, the NNT of avoiding the MACE was not significantly small. But, how far the NNT can represent the significant benefit is not clear. More details of the NNT threshold need to be explored in the future.

## Conclusion

In conclusion, the findings suggest that applying antihypertensive therapy irrespective of the baseline BP and cardiovascular disease status has certain limitations. In our study, people benefited from antihypertensive medication if their SBP was 140 mmHg, and performing BP-lowering treatment for SBPs ranging from 130–140 mmHg might be suitable for subjects with cardiovascular disease or at a high risk of stroke and HF, instead of using a common therapeutic strategy for the whole population. Taking cost-effectiveness into account, it would be more beneficial to target public resources for high-risk patients rather than focusing substantially on healthcare efforts for individuals with low risk and undefined benefits. Our study had some flaws with respect to the participants’ classification, lack of individual data, and unknown NNT thresholds. We carried out analyses solely on the basis of the baseline SBP, not on the different therapies. Therefore, the results might not be representative of all clinical situations in general.

## Data availability statement

The original contributions presented in this study are included in the article/Supplementary material, further inquiries can be directed to the corresponding author.

## Author contributions

Q-BT conceived the study and designed the protocol. YM and SG did the literature search and study selection. YM extracted the relevant information, synthesized and visualized the data, and wrote the first draft of the manuscript. SG verified the data and results. SQ solved controversies about data extraction. All authors had full access to all the data in the study, read and approved the final manuscript, and had final responsibility for the decision to submit for publication.

## Abbreviations

ACC: American College of Cardiology
ACD: all-cause death
AEs: adverse events
ACEI: angiotensin-converting enzyme inhibitor
AHA: American Heart Association
ARB: angiotensin receptor blocker
BMI: body mass index
BP: blood pressure
BPLTTC: Blood Pressure Lowering Treatment Trialists’ Collaboration
CABG: coronary artery bypass grafting
CAD: coronary artery disease
CER: control event rate
CI: confidence interval
CVD: cardiovascular death
DBP: diastolic blood pressure
EER: event rate in treatment group
ESC: European Society of Cardiology
ESH: the European Society of Hypertension
HF: heart failure
HOPE-3: Heart Outcomes Prevention Evaluation-3
ISH: the International Society of Hypertension
MACE: major adverse cardiovascular events
*MeSH*: Medical Subject Headings
MI: myocardial infarction
NNT: the number needed to treat
NNTB: the number needed to be treated for an additional beneficial outcome
NNTH: the number needed to be treated for an additional harmful outcome
PRISMA: Preferred Reporting Items for Systematic Reviews and Meta-Analyses
SBP: systolic blood pressure
WHO: World Health Organization.
