# Corrigendum: Benefits and risks of antihypertensive medication in adults with different systolic blood pressure: A meta-analysis from the perspective of the number needed to treat

**DOI:** 10.3389/fcvm.2022.1094336

**Published:** 2022-12-05

**Authors:** Yucheng Mao, Shiyao Ge, Sufen Qi, Qing-Bao Tian

**Affiliations:** Hebei Key Laboratory of Environment and Human Health, Department of Epidemiology and Statistics, School of Public Health, Hebei Medical University, Shijiazhuang, China

**Keywords:** blood pressure threshold, pharmacological treatment, number needed to treat, cost-effectiveness, cardiovascular endpoint

In the published article, there was an error in Figure 2 as published. The wrong color was used for the visualization of the result in the figure. The numbers of colored faces were inconsistent with some of the results. The corrected Figure 2 and its caption appear below.

**Figure 2:**
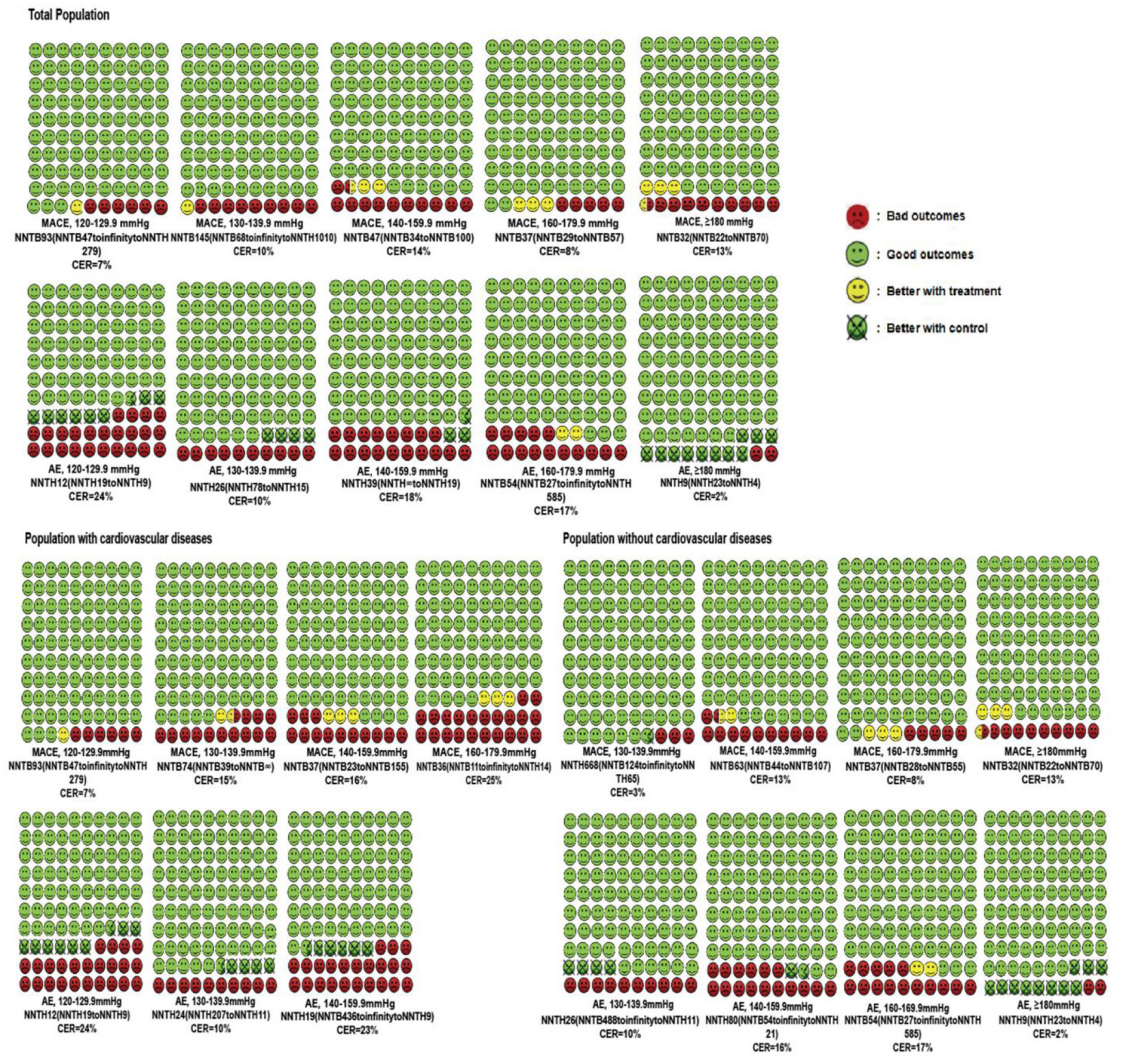
Cates plot of the NNTs for primary outcomes across different SBP levels and cardiovascular diseases status. Each region is the value of NNT of MACEs or AEs in one SBP level and includes 100 faces corresponding to the patients treated with antihypertensive medication. Green faces mean patients not occurring MACEs or AEs with the treatment. Red faces indicate that patients presenting MACEs or AEs with the treatment. Yellow faces represent patients that would not have MACEs or AEs if they would be treated with the treatment. Crossed green faces present patients not fulfilled MACEs or AEs with a control group. Mean follow-up duration was 3.50 years.

In the published article, there was a clerical error in a formula.

A correction has been made to **Materials and methods**, “*Statistical analysis*,” paragraph 2. This formula previously stated:


$$\begin{array}{l} “\text{NNT}=1÷\left(1-\text{relative risk}\right)\times \text{CER}\text{”} \end{array}$$

The corrected formula appears below:


$$\begin{array}{l} “\text{NNT}=1÷\left[\left(1-\text{relative risk}\right)\times \text{CER}\right]\text{”} \end{array}$$

The authors apologize for this error and state that this does not change the scientific conclusions of the article in any way. The original article has been updated.

