# Supplementary material for: Benefits and risks of antihypertensive medication in adults with different systolic blood pressure: A meta-analysis from the perspective of the number needed to treat
Source: Front Cardiovasc Med. 2022 Oct 19;9:986502. doi: 10.3389/fcvm.2022.986502 (PMC9626501; doi:10.3389/fcvm.2022.986502)
Supplement: Supplementary file 1 [file Data_Sheet_1.docx]

**Supplemental material**

**Text summary**

This supplemental material has been provided to give readers additional information including the search strategy, detailed baseline characteristics of included studies, the calculation process of the results and other results or charts that did not represent in the manuscript.

**Contents**

[Method S1. Details of the literature search strategy 3](#_Toc24392)

[Method S2. Detailed inclusion and exclusion criteria 6](#_Toc23639)

[Figure S1. PRISMA Flow Diagram of Study Selection for Quantitative Synthesis 7](#_Toc14755)

[Figure S2. Results of risk assessment 8](#_Toc14818)

[Table S1. Characteristics of studies included in the meta-analysis 9](#_Toc8373)

[Table S2. Detailed computation process of the NNTs for primary outcomes. 12](#_Toc20732)

[Table S3. Detailed computation process of the NNTs for secondary outcomes 14](#_Toc32664)

[Table S4. Data constructing the relationship between the NNT of MACEs and the CER of MACEs 17](#_Toc22370)

[Figure S3. Results of Sensitive Analysis 20](#_Toc2599)

[Table S5. Results of meta-regression 21](#_Toc12130)

[Figure S4. Results of publication bias 22](#_Toc17806)

# Method S1. Details of the literature search strategy

**Data base: PubMed**

**Number of studies: 1618**

1. Mesh=(hypertension)
2. Title/Abstract=(hypertension OR “blood pressure, high” OR “blood pressures, high” OR “high blood pressure” OR “high blood pressures”)
3. #2 OR #1
4. Mesh=(Angiotensin Converting Enzyme Inhibitors)
5. Title/Abstract=(Enzyme Inhibitors, Angiotensin-Converting OR Inhibitors, Angiotensin-Converting Enzyme OR Inhibitors, Angiotensin Converting Enzyme OR Inhibitors, Kininase II OR Kininase II Antagonists OR Kininase II Inhibitors OR Angiotensin-Converting Enzyme Antagonists OR Angiotensin Converting Enzyme Antagonists OR Enzyme Antagonists, Angiotensin-Converting Kininase II Inhibitor OR II Inhibitor, Kininase OR Inhibitor, Kininase II OR Antagonists, Kininase II OR Inhibitors, ACE OR ACE Inhibitors OR Angiotensin I-Converting Enzyme Inhibitors OR Angiotensin I Converting Enzyme Inhibitors OR Angiotensin Converting Enzyme Inhibitor OR ACE Inhibitor OR Inhibitor, ACE OR Angiotensin I-Converting Enzyme Inhibitor OR Angiotensin I Converting Enzyme Inhibitor OR Angiotensin-Converting Enzyme Inhibitor OR Enzyme Inhibitor, Angiotensin-Converting OR Inhibitor, Angiotensin-Converting Enzyme OR Antagonists, Angiotensin-Converting Enzyme OR Antagonists, Angiotensin Converting Enzyme
6. #4 OR #5
7. Mesh=(Angiotensin Receptor Antagonists)
8. Title/Abstract=(Antagonists, Angiotensin Receptor OR Receptor Antagonists, Angiotensin OR Angiotensin Receptor Blockers OR Receptor Blockers, Angiotensin OR Angiotensin Receptor Blocker OR Blocker, Angiotensin Receptor OR Receptor Blocker, Angiotensin OR Angiotensin Receptor Antagonist OR Antagonist, Angiotensin Receptor OR Receptor Antagonist, Angiotensin OR Angiotensin II Receptor Antagonists OR Angiotensin II Receptor Blockers OR Angiotensin II Receptor Antagonist OR Angiotensin II Receptor Blocker)
9. #7 OR #8
10. Mesh= **(**Calcium Channel Blockers)
11. Title/Abstract=( Blockers, Calcium Channel OR Calcium Channel Blocking Drug OR Channel Blockers, Calcium OR Calcium Channel Antagonists OR Antagonists, Calcium Channel OR Calcium Channel Blocking Drugs OR Calcium Channel Antagonist OR Antagonist, Calcium Channel OR Channel Antagonist, Calcium OR Calcium Channel Blocker OR Blocker, Calcium Channel OR Channel Blocker, Calcium OR Calcium Antagonists, Exogenous OR Antagonists, Exogenous Calcium OR Exogenous Calcium Inhibitor OR Calcium Inhibitor, Exogenous OR Inhibitor, Exogenous Calcium OR Exogenous Calcium Inhibitors OR Calcium Inhibitors, Exogenous OR Exogenous Calcium Antagonists OR Exogenous Calcium Blockaders OR Exogenous Calcium Blockader OR Blockader, Exogenous Calcium OR Calcium Blockader, Exogenous OR Calcium Blockaders, Exogenous)
12. #10 OR #11
13. Mesh=(Diuretic)
14. Title/Abstract=(Diuretic Effect OR Effect, Diuretic OR Diuretic Effects OR Effects, Diuretic)
15. #12 OR #13
16. Mesh=( Adrenergic beta-Antagonists)
17. Title/Abstract=( Adrenergic beta Antagonists OR beta-Antagonists, Adrenergic OR Adrenergic beta-Receptor Blockader OR Adrenergic beta Receptor Blockader OR Blockader, Adrenergic beta-Receptor OR beta-Receptor Blockader, Adrenergic OR beta-Adrenergic Antagonist OR Antagonist, beta-Adrenergic OR beta Adrenergic Antagonist OR beta-Adrenergic Blocker OR Blocker, beta-Adrenergic OR beta Adrenergic Blocker OR beta-Adrenergic Antagonists OR Antagonists, beta-Adrenergic OR beta Adrenergic Antagonists OR beta-Adrenoceptor Antagonists OR Antagonists, beta-Adrenoceptor OR beta Adrenoceptor Antagonists OR Adrenergic beta-Receptor Blockaders OR Adrenergic beta Receptor Blockaders OR Blockaders, Adrenergic beta-Receptor OR beta-Receptor Blockaders, Adrenergic OR beta-Adrenergic Receptor Blockaders OR Blockaders, beta-Adrenergic Receptor OR Receptor Blockaders, beta-Adrenergic OR beta Adrenergic Receptor Blockaders OR beta-Adrenergic Blocking Agents OR Agents, beta-Adrenergic Blocking OR Blocking Agents, beta-Adrenergic OR beta Adrenergic Blocking Agents OR beta-Adrenergic Blockers OR Blockers, beta-Adrenergic OR beta Adrenergic Blockers OR beta-Blockers, Adrenergic OR Adrenergic beta-Blockers OR beta Blockers, Adrenergic OR beta-Adrenergic Blocking Agent OR Agent, beta-Adrenergic Blocking OR Blocking Agent, beta-Adrenergic OR beta Adrenergic Blocking Agent OR beta-Adrenergic Receptor Blockader OR Blockader, beta-Adrenergic Receptor OR Receptor Blockader, beta-Adrenergic OR beta Adrenergic Receptor Blockader OR beta-Adrenoceptor Antagonist OR Antagonist, beta-Adrenoceptor OR beta Adrenoceptor Antagonist OR Adrenergic beta-Antagonist OR Adrenergic beta Antagonist OR beta-Antagonist, Adrenergic)
18. #15 OR #16
19. #6 OR #9 OR #12 OR #15 OR #18
20. Title/Abstract = (cardiovascular* OR CV death OR CVD OR all-cause mortality OR mortality OR death* OR myocardial infarction OR heart failure OR stroke OR coronary heart disease OR CHD)
21. #3 AND #19 AND #20
22. Filters: Randomized Controlled Trial OR Clinical Trial, Chinese, English, Human
23. #21 AND #22

**Database: Web of science**

**Number of studies: 3075**

1. TS= (hypertension OR “blood pressure, high” OR “blood pressures, high” OR “high blood pressure” OR “high blood pressures”)

2. TS= (antihypertensive agent* OR angiotensin-converting enzyme inhibitor* OR ACE inhibitor* OR angiotensin II receptor* OR Angiotensin Receptor Blockers OR ARB* OR Calcium Channel Blocker* OR Diuretic* OR beta blocker*)

3. TS= (cardiovascular* OR CV death OR CVD OR all-cause mortality OR mortality OR death* OR myocardial infarction OR heart failure OR stroke OR coronary heart disease OR CHD OR adverse event*)

4. TS= (“randomized control* trial*” OR “clinical trial*” OR “RCT” OR“clinical research*”)

5. (#4 AND #3 AND #2 AND #1)

**Database: Science Direct**

**Number of studies: 377**

Title: hypertension OR “blood pressure, high” OR “blood pressures, high” OR “high blood pressure” OR “high blood pressures”

Title, abstract or keywords: (antihypertensive agent OR angiotensin-converting enzyme inhibitor OR angiotensin II receptor OR Angiotensin Receptor Blockers OR Calcium Channel Blocker OR Diuretic OR beta blocker) AND (randomized control trial OR clinical trial)

Find articles with these terms: CV death OR all-cause mortality OR myocardial infarction OR heart failure OR stroke OR coronary heart disease OR adverse event

**Database: Cochrane Library**

**Number of studies: 6520**

1. Ti/Ab/kw = (hypertension OR blood pressure, high OR blood pressures, high OR high blood pressure OR high blood pressures)

2. Ti/Ab/kw = (antihypertensive agent* OR angiotensin-converting enzyme inhibitor* OR ACE inhibitor* OR angiotensin II receptor* OR Angiotensin Receptor Blockers OR ARB* OR Calcium Channel Blocker* OR Diuretic* OR beta blocker*)

3. Title/Abstract = (cardiovascular* OR CV death OR CVD OR all-cause mortality OR mortality OR death* OR myocardial infarction OR heart failure OR stroke OR coronary heart disease OR CHD)

4. #3 AND #2 AND #1

**Database: Clinical Trials**

**Number of studies: 1744**

Other Terms: randomized

Study Type: Interventional Studies

Condition / Disease: hypertension

Intervention / Treatment: Antihypertensive Agents

# Method S2. Detailed inclusion and exclusion criteria

Inclusion criteria:

1. Randomized assignation to placebo versus five classical anti-hypertensive drugs including Angiotensin Converting Enzyme Inhibitors(ACEI), Angiotensin Receptor Antagonists(ARB), Calcium Channel Blockers(CCB), Adrenergic beta-Antagonists (β-blocker) and Diuretic;
2. Adult participants;
3. The follow-up duration was 1 year or longer in each trial arm;
4. Results reported minimally one of the outcomes of interest (including major cardiovascular events, AEs leading to discontinuation, cardiovascular death, all-cause mortality, heart failure, myocardial infarction, and stroke).

Exclusion criteria:

1. Trials that only included participants with acute settings such as acute myocardial infarction or acute heart failure etc.

# Figure S1. PRISMA Flow Diagram of Study Selection for Quantitative Synthesis

Studies identified through database searching (n=13334)

PubMed (n=1618)

Web of Science (n=3075)

Science Direct (n=377)

The Cochrane Library (n=6520)

Clinical Trial (n=1744)

Duplicates removed automatically (n=2878)

Duplicates removed manually (n=905)

Studies assessed for eligibility in full-text form

(n=66)

After duplicates removed

(n=9551)

Studies excluded after reading the title and abstract (n=9485)

1. Meta-analysis or systematic review or guide or review (n=709)
2. Not prospective randomized controlled study (n=424)
3. Intervention is not one of the 5 classical antihypertensive drugs (n=2291)
4. Acute cardiovascular disease (n=445)
5. Not placebo control (n=2986)
6. Follow-up duration<1y (n=614)
7. No relevant outcomes (n=1677)
8. Participants are not adults (n=19)
9. Publication from the same trial (n=320)

Studies excluded after reading full-test form (n=18)

1. Full-test form is not available (n=3)
2. Publication from the same trial (n=9)
3. Not placebo control (n=4)
4. No relevant outcomes (n=2)

Studies included in this study (n=54)

Studies included in this study (n=48)

Studies searched from other meta-analysis (n=6)

Studies analyzed in this study (n=52)

Studies without information of baseline systolic blood pressure (n=2)

# Figure S2. Results of risk assessment


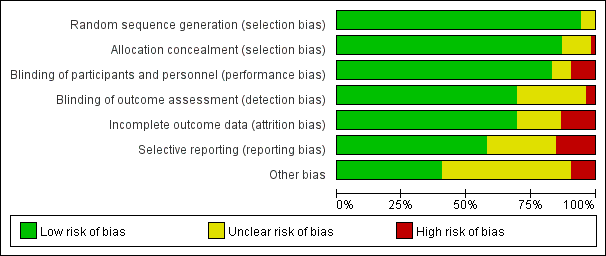


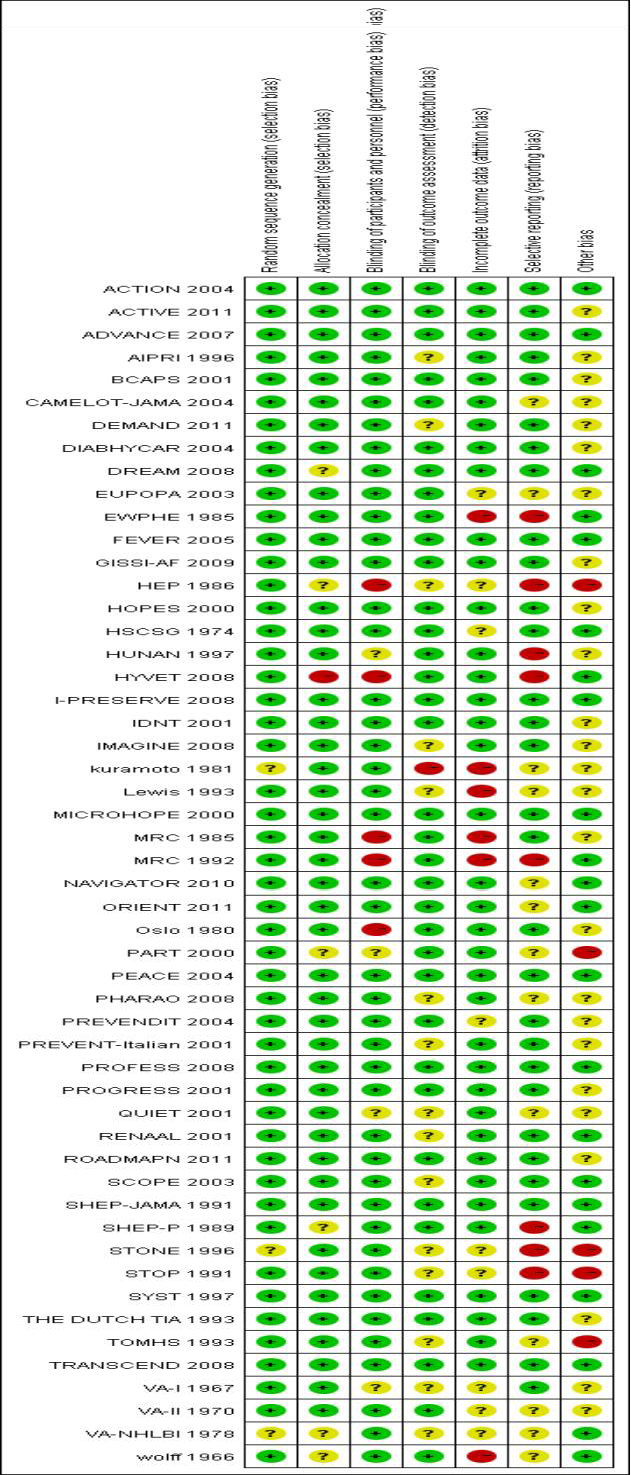


# Table S1. Characteristics of studies included in the meta-analysis

| **Study** | **Year** | **Drug Classification** | **Control** | **population** | | | **Mean Age (years)** | **Female  (%)** | **BMI** | **Hypertension  (%)** | **SBP（mmHg）** | **DBP（mmHg）** | **follow-up (years)** | **Lost to follow-up** | **Cardiovascular diseases status** |
| --- | --- | --- | --- | --- | --- | --- | --- | --- | --- | --- | --- | --- | --- | --- | --- |
|  |  |  |  | **total** | **treatment** | **control** |  |  |  |  |  |  |  |  |  |
| IMAGINE | 2008 | ACEI | placebo | 2553 | 1280 | 1273 | 61 | 13 | NR | 47 | 121.5 | 70 | 2.95 | 5 | Yes |
| QUIET | 2001 | ACEI | placebo | 1750 | 878 | 872 | 58 | 18 | NR | 47 | 123 | 74 | 2.25 | NR | Yes |
| CAMELOT_ACEI | 2004 | ACEI | placebo | 1328 | 673 | 655 | 57.85 | 27.55 | 29.7 | 60 | 129 | 78 | 2 | 38 | Yes |
| CAMELOT_CCB | 2004 | CCB | placebo | 1318 | 663 | 655 | 57.3 | 26 | 29.8 | 60.9 | 129 | 78 | 2 | 34 | Yes |
| PREVENT | 2000 | CCB | placebo | 825 | 417 | 408 | 56.90 | 19.90 | 28.00 | >60% | 129.40 | 78.80 | 3.00 | NR | Yes |
| PREVENDIT | 2004 | ACEI | placebo | 864 | 431 | 433 | 51 | 35 | 26 | NR | 130 | 76 | 3.83 | NR | No |
| PART | 2004 | ACEI | placebo | 617 | 308 | 309 | 61.00 | 18.00 | NR | NR | 133.00 | 79.00 | 4.7 | 1 | Yes |
| PEACE | 2004 | ACEI | placebo | 8290 | 4158 | 4132 | 64 | 18 | NR | 45.5 | 133 | 78 | 4.8 | 134 | Yes |
| PHARAO | 2008 | ACEI | placebo | 1008 | 505 | 503 | 62.25 | 51.59 | 26.85 | NR | 134.4 | 83.6 | 3 | 135 | No |
| ROADMAP | 2011 | ARB | placebo | 4299 | 2160 | 2139 | 57.70 | 53.90 | 31 | 82 | 136 | 81 | 3.2 | NR | No |
| I-PRESERVE | 2008 | ARB | placebo | 4128 | 2067 | 2061 | 66.90 | 43.00 | 29.65 | 76.40 | 136.50 | 79.00 | 4.67 | 18 | Yes |
| EUROPA | 2003 | ACEI | placebo | 12218 | 6110 | 6108 | 60 | 15 | NR | 27.1 | 137 | 82 | 4.2 | NR | Yes |
| ACTION | 2004 | CCB | placebo | 7665 | 3825 | 3840 | 63.5 | 20.5 | NR | 52 | 137.5 | 79.9 | 4.9 | NR | Yes |
| DREAM | 2008 | ACEI | placebo | 5269 | 2623 | 2646 | 56.6 | 41.8 | 30.9 | 48.9 | 137.6 | 83.5 | 3 | NR | No |
| ACTIVE | 2011 | ARB | placebo | 9016 | 4518 | 4498 | 69.60 | 39.30 | 28.80 | 88.00 | 138.30 | 82.60 | 4.10 | NR | Yes |
| Lewis | 1993 | ACEI | placebo | 409 | 207 | 202 | 34.50 | 47.00 | NR | 75.5 | 138.50 | 85.50 | 3 | NR | No |
| GISSI-AF | 2009 | ARB | placebo | 1442 | 722 | 720 | 67.90 | 37.75 | 27.85 | 85.4 | 138.60 | 81.60 | 1 | 10 | Yes |
| BCAPS | 2001 | CCB | placebo | 793 | 396 | 397 | 61.80 | 54.50 | 25.5 | 12.2 | 138.9 | 84.7 | 2.99 | NR | No |
| HOPES | 2000 | ACEI | placebo | 9297 | 4645 | 4652 | 66 | 27 | 28 | 47.4 | 139 | 79 | 5 | NR | Yes |
| NAVIGATOR | 2010 | ARB | placebo | 9306 | 4631 | 4675 | 63.80 | 50.70 | 30.5 | 77.55 | 139.70 | 82.60 | 5 | 1833 | No |
| TOMHS | 1993 | β-bloker | placebo | 902 | 668 | 234 | 54.8 | 34.8 | NR | 100 | 140.4 | 90.5 | 4.4 | NR | No |
| TRANSCEND | 2008 | ARB | placebo | 5926 | 2954 | 2972 | 66.90 | 60.00 | 28.20 | 76.40 | 141.00 | 81.90 | 4.67 | 18 | Yes |
| ORIENT | 2011 | ARB | placebo | 566 | 282 | 284 | 59.2 | 30.9 | 25.3 | 93.8 | 141.3 | 77.5 | 3.2 | NR | No |
| MICRO-HOPE | 2000 | ACEI | placebo | 3577 | 1808 | 1769 | 65.5 | 36.5 | 28.8 | 56 | 142 | 79.65 | 4.5 | NR | Yes |
| AIPRI | 1996 | ACEI | placebo | 583 | 300 | 283 | 51.00 | 28.00 | NR | 82 | 143 | 87.5 | 3 | NR | No |
| PROGRESS_ACEI | 2001 | ACEI | placebo | 2561 | 1281 | 1280 | 65 | 32 | NR | 40 | 144 | 84 | 3.9 | NR | Yes |
| PRoFESS | 2008 | ARB | placebo | 20332 | 10146 | 10186 | 66.20 | 36.00 | 26.80 | 74.00 | 144.10 | 83.80 | 2.50 | 125 | Yes |
| ADVANCE | 2007 | ACEI+CCB | placebo | 11140 | 5569 | 5571 | 66 | 39.27 | 28 | 68.5 | 145 | 81 | 4.3 | 15 | No |
| DIABHYCAR | 2004 | ACEI | placebo | 4912 | 2443 | 2469 | 65.1 | 30.13 | 29.2 | 55.7 | 145.45 | 82.3 | 4 | 160 | No |
| DEMAND_ACEI | 2011 | ACEI | placebo | 254 | 127 | 127 | 61.2 | 33.1 | 29.6 | 100 | 147.1 | 87.2 | 3.8 | NR | No |
| DEMAND_CCB+ACEI | 2011 | CCB+ACEI | placebo | 253 | 126 | 127 | 60.3 | 34.4 | 29.6 | 100 | 147.8 | 87.2 | 3.8 | NR | No |
| PROGRESS_combination | 2001 | ACEI+Diuretic | placebo | 3544 | 1770 | 1774 | 63 | 29 | NR | 54 | 149 | 87 | 3.9 | NR | Yes |
| RENAAL | 2001 | ARB | placebo | 1513 | 751 | 762 | 60.00 | 36.85 | 29.5 | 93 | 152.5 | NR | 3.4 | NR | No |
| FEVER | 2005 | CCB | placebo | 9711 | 4841 | 4870 | 61.50 | 38.85 | 26.25 | 100 | 154.30 | 91.15 | 3.33 | 30 | No |
| Oslo | 1980 | Diuretic+central | placebo | 785 | 406 | 379 | NR | 0 | NR | 100 | 155.75 | 96.8 | 5.5 | NR | No |
| Dutch TIA | 1993 | β-bloker | placebo | 1473 | 732 | 741 | NR | 91.30 | NR | 100.00 | 157.50 | 91.00 | 2.60 | 0 | Yes |
| IDNT-CCB | 2001 | CCB | placebo | 1136 | 567 | 569 | 58.70 | 32.92 | 30.70 | 100.00 | 158.50 | 87.00 | 2.60 | 6 | No |
| IDNT-ARB | 2001 | ARB | placebo | 1148 | 579 | 569 | 58.80 | 31.97 | 30.80 | 100.00 | 159.00 | 87.00 | 2.60 | 9 | No |
| VA-NHLBI | 1978 | Diuretic+CCB | placebo | 1012 | 508 | 504 | 37.5 | 19 | NR | 100 | 160 | 95 | 1 | NR | No |
| HUNAN | 1997 | CCB | placebo | 2080 | 1040 | 1040 | 51.8 | 47 | NR | 100 | 160.5 | 98.51 | 4.72 | NR | No |
| MRC_mild_Diuretic | 1985 | Diuretic | placebo | 12951 | 4297 | 8654 | 52.00 | 47.86 | NR | 100 | 161.50 | 97.00 | 5.5 | NR | No |
| MRC_mild_β-blocker | 1985 | β-bloker | placebo | 13057 | 4403 | 8654 | 52.00 | 48.86 | NR | 100 | 161.50 | 97.00 | 5.5 | NR | No |
| VA-II | 1970 | Diuretic+CCB | placebo | 380 | 186 | 194 | 51.25 | 0 | 26.8 | 100 | 163.6 | 104.25 | 3.25 | 56 | No |
| SCOPE | 2003 | ARB | placebo | 4937 | 2477 | 2460 | 76.4 | 64.5 | 27 | 52.7 | 166.25 | 90.35 | 3.7 | 8 | No |
| HSCSG | 1974 | Diuretic+CCB | placebo | 452 | 226 | 226 | 59 | 40 | NR | 100 | 167 | 100 | 2.75 | NR | Yes |
| Kuramoto | 1981 | Diuretic+CCB+central | placebo | 79 | 38 | 41 | 76.1 | NR | NR | 100 | 168.7 | 86 | 4 | NR | No |
| STONE | 1996 | CCB | placebo | 1666 | 838 | 828 | 66.42 | 53.12 | 24.3 | 100 | 169 | 98 | 2.5 | 131 | No |
| SHEP | 1991 | Diuretic+β-bloker | placebo | 4736 | 2365 | 2371 | 72 | 57 | 27.5 | 100 | 170.3 | 76.6 | 4.5 | NR | No |
| SHEP-P | 1989 | Diuretic | placebo | 551 | 443 | 108 | 72.1 | 63 | NR | 100 | 172 | 75 | 2.83 | NR | No |
| HYVET | 2008 | Diuretic | placebo | 3845 | 1933 | 1912 | 83.60 | 60.50 | 24.70 | 89.90 | 173.00 | 90.80 | 2.10 | 17 | No |
| Syst-Eur | 1997 | CCB+(ACEI+Diuretic) | placebo | 4695 | 2398 | 2297 | 70.3 | 66.85 | 26.9 | 100 | 173.85 | 85.5 | 2 | 237 | No |
| Wolff | 1966 | CCB+Diuretic | placebo | 87 | 45 | 42 | 43.5 | 67.82 | NR | 100 | 176.7 | 108.9 | 2 | NR | No |
| EWPHE | 1985 | Diuretic | placebo | 840 | 416 | 424 | 72 | 69 | NR | 100 | 182.5 | 101 | 4.7 | 128 | No |
| MRC-old_Diuretic | 1992 | Diuretic | placebo | 3294 | 1081 | 2213 | 70.30 | 58.11 | 26.48 | 100 | 184.50 | 90.75 | 5.8 | NR | No |
| MRC-old_β-bloker | 1992 | β-bloker | placebo | 3315 | 1102 | 2213 | 70.30 | 58.31 | 26.55 | 100 | 184.50 | 90.75 | 5.8 | NR | No |
| VA-I | 1967 | Diuretic+CCB+hydralazine hydrochloride | placebo | 143 | 73 | 70 | 50.7 | 0 | NR | 100 | 186.2 | 121.1 | 1.5 | NR | No |
| STOP | 1991 | β-blocker+Diuretic | placebo | 1627 | 812 | 815 | 75.7 | 63 | 26.6 | 100 | 195 | 102 | 2.08 | NR | No |
| HEP | 1986 | β-blocker+Diuretic | placebo | 884 | 419 | 465 | NR | 69.5 | NR | 100 | 196.15 | 98.6 | 4.4 | NR | No |

**Abbreviations:** ACEI, angiotensin-converting enzyme inhibitors; ARB, angiotensin II receptor blocker; CCB, calcium-channel block; β-blocker, beta-adrenergic receptor blocker; BMI, body mass index; SBP, systolic blood pressure; DBP, diastolic blood pressure; follow-up, follow-up duration in years; NR, not report.

# Table S2. Detailed computation process of the NNTs for primary outcomes

| **Population** | **Primary Outcomes** | **Baseline SBP** | **Trials(n)** | **Pooled RR(95%CI)** | **Pooled CER** | **NNT(95%CI)** | ***I^2^*** | **follow-up years** | **Y-S** | **NNT_S** | **CER_S** |
| --- | --- | --- | --- | --- | --- | --- | --- | --- | --- | --- | --- |
| Total population | MACE | 120-129.9mmHg | 5 | 0.85(0.70,1.05) | 0.05 | NNTB134(NNTB67toinfinitytoNNTH400) | 0.00% | 2.4400 | 3.50 | NNTB93(NNTB47toinfinitytoNNTH279) | 0.07 |
|  |  | 130-139.9mmHg | 14 | 0.93(0.85,1.01) | 0.11 | NNTB130(NNTB61toinfinitytoNNTH910) | 72.80% | 3.8850 | 3.50 | NNTB145(NNTB68toinfinitytoNNTH1010) | 0.10 |
|  |  | 140-159.9mmHg | 18 | 0.85(0.79,0.93) | 0.15 | NNTB45(NNTB32toNNTB96) | 67.60% | 3.6700 | 3.50 | NNTB47(NNTB34toNNTB100) | 0.14 |
|  |  | 160-179.9mmHg | 14 | 0.68(0.59,0.79) | 0.08 | NNTB40(NNTB31toNNTB60) | 57.30% | 3.3100 | 3.50 | NNTB37(NNTB29toNNTB57) | 0.08 |
|  |  | ≥180mmHg | 5 | 0.76(0.64,0.89) | 0.17 | NNTB25(NNTB17toNNTB54) | 53.20% | 4.5560 | 3.50 | NNTB32(NNTB22toNNTB70) | 0.13 |
|  | AE | 120-129.9mmHg | 3 | 1.35(1.22,1.50) | 0.16 | NNTH18(NNTH29toNNTH13) | 0.00% | 2.3200 | 3.50 | NNTH12(NNTH19toNNTH9) | 0.24 |
|  |  | 130-139.9mmHg | 7 | 1.39(1.13,1.17) | 0.10 | NNTH26(NNTH77toNNTH15) | 85.60% | 3.5400 | 3.50 | NNTH26(NNTH78toNNTH15) | 0.10 |
|  |  | 140-159.9mmHg | 8 | 1.14(1.00,1.30) | 0.18 | NNTH40(NNTH∞toNNTH19) | 82.80% | 3.4375 | 3.50 | NNTH39(NNTH∞toNNTH19) | 0.18 |
|  |  | 160-179.9mmHg | 2 | 0.89(0.78,1.01) | 0.17 | NNTB54(NNTB27toinfinitytoNNTH589) | 24.20% | 3.4750 | 3.50 | NNTB54(NNTB27toinfinitytoNNTH585) | 0.17 |
|  |  | ≥180mmHg | 2 | 5.72(2.84,11.51) | 0.04 | NNTH6(NNTH14toNNTH3) | 93.90% | 5.8000 | 3.50 | NNTH9(NNTH23toNNTH4) | 0.02 |
| Population with cardiovascular diseases | MACE | 120-129.9mmHg | 5 | 0.85(0.70,1.05) | 0.05 | NNTB134(NNTB67toinfinitytoNNTH400) | 0.00% | 2.4400 | 3.50 | NNTB93(NNTB47toinfinitytoNNTH279) | 0.07 |
|  |  | 130-139.9mmHg | 8 | 0.91(0.83,1.00) | 0.18 | NNTB62(NNTB33toNNTB∞） | 80.60% | 4.1700 | 3.50 | NNTB74(NNTB39toNNTB∞) | 0.15 |
|  |  | 140-159.9mmHg | 6 | 0.83(0.72,0.96) | 0.17 | NNTB35(NNTB22toNNTB148) | 84.50% | 3.6800 | 3.50 | NNTB37(NNTB23toNNTB155) | 0.16 |
|  |  | 160-179.9mmHg | 1 | 0.89(0.61(1.30) | 0.20 | NNTB46(NNTB13toinfinitytoNNTH17) | 0.00% | 2.7500 | 3.50 | NNTB36(NNTB11toinfinitytoNNTH14) | 0.25 |
|  | AE | 120-129.9mmHg | 3 | 1.35(1.22,1.50) | 0.16 | NNTH18(NNTH29toNNTH13) | 0.00% | 2.3200 | 3.50 | NNTH12(NNTH19toNNTH9) | 0.24 |
|  |  | 130-139.9mmHg | 3 | 1.44(1.05,1.98) | 0.09 | NNTH26(NNTH223toNNTH12) | 81.40% | 3.2600 | 3.50 | NNTH24(NNTH207toNNTH11) | 0.10 |
|  |  | 140-159.9mmHg | 3 | 1.23(0.99,1.52) | 0.21 | NNTH21(NNTB477toinfinitytoNNTH10) | 91.60% | 3.2000 | 3.50 | NNTH19(NNTB436toinfinitytoNNTH9) | 0.23 |
| Population without cardiovascular diseases | MACE | 130-139.9mmHg | 6 | 1.05(0.73,1.52) | 0.03 | NNTH667(NNTB124toinfinitytoNNTH65) | 51.40% | 3.5030 | 3.50 | NNTH668(NNTB124toinfinitytoNNTH65) | 0.03 |
|  |  | 140-159.9mmHg | 12 | 0.88(0.83,0.93) | 0.14 | NNTB60(NNTB43toNNTB103) | 43.60% | 3.6610 | 3.50 | NNTB63(NNTB44toNNTB107) | 0.13 |
|  |  | 160-179.9mmHg | 13 | 0.67(0.57,0.78) | 0.08 | NNTB38(NNTB30toNNTB57) | 59.30% | 3.3540 | 3.50 | NNTB37(NNTB28toNNTB55) | 0.08 |
|  |  | ≥180mmHg | 5 | 0.76(0.64,0.89) | 0.17 | NNTB25(NNTB17toNNTB54) | 53.20% | 4.5560 | 3.50 | NNTB32(NNTB22toNNTB70) | 0.13 |
|  | AEs | 130-139.9mmHg | 4 | 1.38(0.98,1.94) | 0.11 | NNTH24(NNTB455toinfinitytoNNTH10) | 89.70% | 3.7550 | 3.50 | NNTH26(NNTB488toinfinitytoNNTH11) | 0.10 |
|  |  | 140-159.9mmHg | 5 | 1.08(0.88,1.31) | 0.16 | NNTH79(NNTB53toinfinitytoNNTH21) | 69.00% | 3.5800 | 3.50 | NNTH80(NNTB54toinfinitytoNNTH21) | 0.16 |
|  |  | 160-179.9mmHg | 2 | 0.89(0.78,1.01) | 0.17 | NNTB54(NNTB27toinfinitytoNNTH589) | 24.20% | 3.4750 | 3.50 | NNTB54(NNTB27toinfinitytoNNTH585) | 0.17 |
|  |  | ≥180mmHg | 2 | 5.72(2.84,11.51) | 0.04 | NNTH6(NNTH14toNNTH3) | 93.90% | 5.8000 | 3.50 | NNTH9(NNTH23toNNTH4) | 0.02 |

**Abbreviations:** NNT, the number needed to treat; NNTB, the number needed to treat for an additional beneficial outcome; NNTH, the number needed to treat for an additional harmful outcome; CER, control events rate; Y-S, the ratio of the observed follow-up duration to the mean follow-up duration among the included trials; NNT_S, the NNT after standardization; CER_S, the control events rate after standardization; MACEs, major adverse cardiovascular events; AEs, adverse events leading to discontinuation.

# Table S3. Detailed computation process of the NNTs for secondary outcomes

| **Population** | **Secondary outcomes** | **Baseline SBP** | **Trials(n)** | **Pooled RR(95%CI)** | **Pooled CER** | **NNT(95%CI)** | **I**2 | **mean follow-up years** | **Y-S** | **NNT_S** |
| --- | --- | --- | --- | --- | --- | --- | --- | --- | --- | --- |
| Total population | CVD | 120-129.9mmHg | 5 | 1.13(0.72,1.77) | 0.01 | NNTH770(NNTB358toinfinitytoNNTH130) | 0.00% | 2.4400 | 3.50 | NNTH537(NNTB249toinfinitytoNNTH91) |
|  |  | 130-139.9mmHg | 12 | 0.96(0.86,1.07) | 0.06 | NNTB417(NNTB120toinfinitytoNNTH239) | 59.60% | 4.0300 | 3.50 | NNTB480(NNTB138toinfinitytoNNTH275) |
|  |  | 140-159.9mmHg | 15 | 0.89(0.81,0.99) | 0.10 | NNTB91(NNTB53toNNTB1000) | 54.90% | 3.6800 | 3.50 | NNTB96(NNTB56toNNTB1052) |
|  |  | 160-179.9mmHg | 8 | 0.82(0.73,0.93) | 0.05 | NNTB112(NNTB75toNNTB286) | 0.00% | 3.1100 | 3.50 | NNTB99(NNTB66toNNTB254) |
|  |  | ≥180mmHg | 5 | 0.84(0.73,0.97) | 0.10 | NNTB63(NNTB38toNNTB334) | 31.70% | 4.5560 | 3.50 | NNTB82(NNTB49toNNTB434) |
|  | ACD | 120-129.9mmHg | 5 | 1.00(0.73,1.38) | 0.02 | NNTB∞(NNTB186toinfinitytoNNTH132) | 0.00% | 2.4400 | 3.50 | NNTB∞(NNTB130toinfinitytoNNTH92) |
|  |  | 130-139.9mmHg | 14 | 0.95(0.90,0.99) | 0.07 | NNTB286(NNTB143toNNTB1429) | 32.10% | 3.8260 | 3.50 | NNTB313(NNTB157toNNTB1562) |
|  |  | 140-159.9mmHg | 12 | 0.96(0.91,1.01) | 0.10 | NNTB250(NNTB112toinfinitytoNNTH1000) | 43.70% | 3.3920 | 3.50 | NNTB243(NNTB108toinfinitytoNNTH970) |
|  |  | 160-179.9mmHg | 12 | 0.90(0.84,0.98) | 0.07 | NNTB143(NNTB90toNNTB715) | 0.00% | 3.3625 | 3.50 | NNTB138(NNTB86toNNTB687) |
|  |  | ≥180mmHg | 6 | 0.89(0.76,1.05) | 0.15 | NNTB61(NNTB28toinfinitytoNNTH134) | 53.10% | 4.0470 | 3.50 | NNTB71(NNTB33toinfinitytoNNTH155) |
|  | MI | 120-129.9mmHg | 5 | 0.80(0.61,1.04) | 0.03 | NNTB167(NNTB86toinfinitytoNNTH834) | 0.00% | 2.4400 | 3.50 | NNTB117(NNTB60toinfinitytoNNTH581) |
|  |  | 130-139.9mmHg | 12 | 0.91(0.85,0.97) | 0.04 | NNTB278(NNTB167toNNTB834) | 47.10% | 4.1825 | 3.50 | NNTB332(NNTB200toNNTB996) |
|  |  | 140-159.9mmHg | 8 | 0.83(0.75,0.93) | 0.05 | NNTB118(NNTB80toNNTB286) | 0.00% | 3.8460 | 3.50 | NNTB130(NNTB88toNNTB314) |
|  |  | 160-179.9mmHg | 9 | 0.83(0.68,1.02) | 0.02 | NNTB295(NNTB157toinfinitytoNNTH2500) | 0.00% | 2.8200 | 3.50 | NNTB237(NNTB126toinfinitytoNNTH2015) |
|  |  | ≥180mmHg | 2 | 0.73(0.47,1.15) | 0.04 | NNTB93(NNTB48toinfinitytoNNTH167) | 43.80% | 3.3900 | 3.50 | NNTB90(NNTB46toinfinitytoNNTH162) |
|  | HF | 120-129.9mmHg | 4 | 0.78(0.45,1.35) | 0.01 | NNTB455(NNTB182toinfinitytoNNTH286) | 0.00% | 2.4875 | 3.50 | NNTB324(NNTB130toinfinitytoNNTH204) |
|  |  | 130-139.9mmHg | 9 | 0.86(0.80,0.93) | 0.05 | NNTB143(NNTB100toNNTB286) | 43.30% | 4.1560 | 3.50 | NNTB170(NNTB119toNNTB340) |
|  |  | 140-159.9mmHg | 7 | 0.88(0.79,0.99) | 0.04 | NNTB209(NNTB120toNNTB2500) | 39.80% | 3.6570 | 3.50 | NNTB218(NNTB125toNNTB2613) |
|  |  | 160-179.9mmHg | 6 | 0.47(0.23,0.95) | 0.03 | NNTB63(NNTB44toNNTB667) | 68.20% | 2.4300 | 3.50 | NNTB44(NNTB31toNNTB463) |
|  | Stroke | 120-129.9mmHg | 12 | 0.83(0.77,0.91) | 0.03 | NNTB197(NNTB145toNNTB371) | 27.00% | 3.9470 | 3.50 | NNTB324(NNTB143toinfinitytoNNTH324) |
|  |  | 130-139.9mmHg | 11 | 0.81(0.70,0.93) | 0.06 | NNTB88(NNTB56toNNTB239) | 67.30% | 3.7360 | 3.50 | NNTB222(NNTB164toNNTB418) |
|  |  | 140-159.9mmHg | 11 | 0.63(0.56,0.71) | 0.05 | NNTB55(NNTB46toNNTB69) | 42.40% | 3.4640 | 3.50 | NNTB94(NNTB60toNNTB255) |
|  |  | 160-179.9mmHg | 4 | 0.69(0.58,0.83) | 0.07 | NNTB47(NNTB35toNNTB85) | 3.40% | 4.5200 | 3.50 | NNTB54(NNTB45toNNTB69) |
|  |  | ≥180mmHg | 5 | 1.13(0.72,1.77) | 0.01 | NNTH770(NNTB358toinfinitytoNNTH130) | 0.00% | 2.4400 | 3.50 | NNTB60(NNTB44toNNTB109) |
| Population with cardiovascular diseases | CVD | 120-129.9mmHg | 5 | 1.13(0.72,1.77) | 0.01 | NNTH770(NNTB358toinfinitytoNNTH130) | 0.00% | 2.4400 | 3.50 | NNTH537(NNTB249toinfinitytoNNTH91) |
|  |  | 130-139.9mmHg | 8 | 0.92(0.83,1.02) | 0.09 | NNTB139(NNTB66toinfinitytoNNTH556) | 60.80% | 4.1700 | 3.50 | NNTB166(NNTB78toinfinitytoNNTH662) |
|  |  | 140-159.9mmHg | 4 | 0.88(0.70,1.12) | 0.06 | NNTB139(NNTB56toinfinitytoNNTH139) | 77.00% | 3.5675 | 3.50 | NNTB142(NNTB57toinfinitytoNNTH142) |
|  | ACD | 120-129.9mmHg | 5 | 1.00(0.73,1.38) | 0.02 | NNTB∞(NNTB186toinfinitytoNNTH132) | 0.00% | 2.4400 | 3.50 | NNTB∞(NNTB130toinfinitytoNNTH92) |
|  |  | 130-139.9mmHg | 8 | 0.95(0.90,1.00) | 0.09 | NNTB223(NNTB112toNNTB∞) | 41.30% | 4.1700 | 3.50 | NNTB265(NNTB133toNNTB∞) |
|  |  | 140-159.9mmHg | 4 | 0.97(0.84,1.12) | 0.10 | NNTB334(NNTB63toinfinitytoNNTH84) | 67.60% | 3.5675 | 3.50 | NNTB340(NNTB64toinfinitytoNNTH85) |
|  | MI | 120-129.9mmHg | 5 | 0.80(0.61,1.04) | 0.03 | NNTB167(NNTB86toinfinitytoNNTH834) | 0.00% | 2.4400 | 3.50 | NNTB117(NNTB60toinfinitytoNNTH581) |
|  |  | 130-139.9mmHg | 7 | 0.94(0.82(1.07) | 0.06 | NNTB278(NNTB93toinfinitytoNNTH239) | 68.10% | 4.6200 | 3.50 | NNTB367(NNTB123toinfinitytoNNTH315) |
|  |  | 140-159.9mmHg | 3 | 0.86(0.76,0.96) | 0.06 | NNTB120(NNTB70toNNTB417) | 36.10% | 3.8900 | 3.50 | NNTB133(NNTB78toNNTB464) |
|  |  | 160-179.9mmHg | 1 | 1.00(0.25,3.95) | 0.02 | NNTB∞(NNTB67toinfinitytoNNTH17) | 0.00% | 2.7500 | 3.50 | NNTB∞(NNTB53toinfinitytoNNTH14) |
|  | HF | 120-129.9mmHg | 4 | 0.78(0.45,1.35) | 0.01 | NNTB455(NNTB182toinfinitytonNTH286) | 0.00% | 2.4875 | 3.50 | NNTB324(NNTB130toinfinitytoNNTH204) |
|  |  | 130-139.9mmHg | 5 | 0.84(0.78,0.92) | 0.07 | NNTB90(NNTB65toNNTB179) | 42.70% | 4.5140 | 3.50 | NNTB116(NNTB84toNNTB231) |
|  |  | 140-159.9mmHg | 3 | 1.03(0.89,1.20) | 0.03 | NNTH1112(NNTB304toinfinitytoNNTH167) | 0.00% | 3.8900 | 3.50 | NNTH1235(NNTB337toinfinitytoNNTH186) |
|  |  | 160-179.9mmHg | 1 | 0.08(0.00,1.36) | 0.03 | NNTB37(NNTB34toinfinitytoNNTH93) | 0.00% | 2.7500 | 3.50 | NNTB29(NNTB27toinfinitytoNNTH73) |
|  | Stroke | 120-129.9mmHg | 4 | 0.78(0.50,1.22) | 0.01 | NNTB455(NNTB200toinfinitytoNNTH455) | 0.00% | 2.4875 | 3.50 | NNTB324(NNTB143toinfinitytoNNTH324) |
|  |  | 130-139.9mmHg | 8 | 0.84(0.77,0.92) | 0.04 | NNTB157(NNTB109toNNTB313) | 36.30% | 4.1700 | 3.50 | NNTB187(NNTB130toNNTB373) |
|  |  | 140-159.9mmHg | 6 | 0.80(0.67,0.96) | 0.09 | NNTB56(NNTB34toNNTB278) | 78.20% | 3.6780 | 3.50 | NNTB59(NNTB36toNNTB292) |
|  |  | 160-179.9mmHg | 1 | 0.88(0.59,1.32) | 0.19 | NNTB44(NNTB13toinfinitytoNNTH17) | 0.00% | 2.7500 | 3.50 | NNTB35(NNTB11toinfinitytoNNTH13) |
| Population without cardiovascular diseases | CVD | 130-139.9mmHg | 4 | 1.22(0.97,1.54) | 0.01 | NNTH455(NNTB3334toinfinitytoNNTH186) | 46.50% | 3.7575 | 3.50 | NNTH488(NNTB3579toinfinitytoNNTH199) |
|  |  | 140-159.9mmHg | 11 | 0.91(0.85,0.98) | 0.12 | NNTB93(NNTB56toNNTB417) | 47.90% | 3.7210 | 3.50 | NNTB99(NNTB60toNNTB443) |
|  |  | 160-179.9mmHg | 8 | 0.82(0.73,0.93) | 0.05 | NNTB112(NNTB75toNNTB286) | 0.00% | 3.1100 | 3.50 | NNTB99(NNTB66toNNTB254) |
|  |  | ≥180mmHg | 5 | 0.84(0.73,0.97) | 0.10 | NNTB63(NNTB38toNNTB334) | 31.70% | 4.5560 | 3.50 | NNTB82(NNTB49toNNTB434） |
|  | ACD | 130-139.9mmHg | 6 | 0.94(0.82,1.07) | 0.03 | NNTB556(NNTB186toinfinitytoNNTH477) | 30.00% | 3.3650 | 3.50 | NNTB535(NNTB179toinfinitytoNNTH458) |
|  |  | 140-159.9mmHg | 8 | 0.92(0.86,0.99) | 0.10 | NNTB125(NNTB72toNNTB1000) | 20.80% | 3.3040 | 3.50 | NNTB118(NNTB68toNNTB944) |
|  |  | 160-179.9mmHg | 12 | 0.90(0.84,0.98) | 0.07 | NNTB143(NNTB90toNNTB715) | 0.00% | 3.3625 | 3.50 | NNTB138(NNTB86toNNTB687) |
|  |  | ≥180mmHg | 6 | 0.89(0.76,1.05) | 0.15 | NNTB61(NNTB28toinfinitytoNNTH134) | 53.10% | 4.0500 | 3.50 | NNTB71(NNTB33toinfinitytoNNTH155) |
|  | MI | 130-139.9mmHg | 5 | 1.00(0.81,1.24) | 0.02 | NNTB∞(NNTB264toinfinitytoNNTH209) | 0.00% | 3.5640 | 3.50 | NNTB∞(NNTB268toinfinitytoNNTH213) |
|  |  | 140-159.9mmHg | 5 | 0.77(0.61,0.96) | 0.03 | NNTB145(NNTB86toNNTB834) | 0.00% | 3.8200 | 3.50 | NNTB159(NNTB94toNNTB910) |
|  |  | 160-179.9mmHg | 8 | 0.83(0.68,1.01) | 0.02 | NNTB295(NNTB157toinfinitytoNNTH5000) | 0.00% | 2.8290 | 3.50 | NNTB238(NNTB127toinfinitytoNNTH4042) |
|  |  | ≥180mmHg | 2 | 0.73(0.47,1.15) | 0.04 | NNTB93(NNTB48toinfinitytoNNTH167) | 43.80% | 3.3900 | 3.50 | NNTB90(NNTB46toinfinitytoNNTH162) |
|  | HF | 130-139.9mmHg | 4 | 1.02(0.80,1.32) | 0.02 | NNTH2500(NNTB250toinfinitytoNNTH157) | 46.40% | 3.7075 | 3.50 | NNTH2649(NNTB265toinfinitytoNNTH166) |
|  |  | 140-159.9mmHg | 4 | 0.76(0.64,0.90) | 0.07 | NNTB60(NNTB40toNNTB143) | 0.00% | 3.4825 | 3.50 | NNTB60(NNTB40toNNTB143) |
|  |  | 160-179.9mmHg | 5 | 0.48(0.35,0.65) | 0.02 | NNTB97(NNTB77toNNTB143) | 49.70% | 2.3700 | 3.50 | NNTB66(NNTB53toNNTB97) |
|  | Stroke | 130-139.9mmHg | 4 | 0.76(0.60,0.97) | 0.01 | NNTB417(NNTB250toNNTB3334) | 18.30% | 3.4975 | 3.50 | NNTB417(NNTB250toNNTB3331) |
|  |  | 140-159.9mmHg | 5 | 0.80(0.69,0.93) | 0.03 | NNTB167(NNTB108toNNTB477) | 42.40% | 3.8060 | 3.50 | NNTB182(NNTB117toNNTB518) |
|  |  | 160-179.9mmHg | 10 | 0.61(0.54,0.69) | 0.04 | NNTB65(NNTB55toNNTB81) | 38.90% | 3.5350 | 3.50 | NNTB65(NNTB55toNNTB82) |
|  |  | ≥180mmHg | 4 | 0.69(0.58,0.83) | 0.07 | NNTB47(NNTB35toNNTB85) | 3.40% | 4.5200 | 3.50 | NNTB60(NNTB44toNNTB109) |

# Abbreviations: NNT, the number needed to treat; NNTB, the number needed to treat for an additional beneficial outcome; NNTH, the number needed to treat for an additional harmful outcome; CER, control events rate; Y-S, the ratio of the observed follow-up duration to the mean follow-up duration among the included trials; NNT_S, the NNT after standardization; CER_S, the control events rate after standardization; CVD, cardiovascular death; ACD, all-cause death; MI, myocardial infarction; HF, heart failure.

# Table S4. Data constructing the relationship between the NNT of MACEs and the CER of MACEs

| **Study** | **Base_SBP（mmHg）** | **a** | **b** | **d** | **n1** | **n2** | **n** | **CER** | **Follow-up,y** | **NNT** | **NNT_CL** | **NNT_UL** | **Y-S** | **NNT_S** | **NNT_S_CL** | **NNT_S_UL** |
| --- | --- | --- | --- | --- | --- | --- | --- | --- | --- | --- | --- | --- | --- | --- | --- | --- |
| ROADMAP | 136 | 15 | 2217 | 2212 | 2232 | 2215 | 4447 | 0.00 | 3.2 | -186.36 | -607.35 | -110.06 | 3.60 | -165.65 | -539.87 | -97.84 |
| VA-NHLBI | 160 | 6 | 502 | 499 | 508 | 504 | 1012 | 0.01 | 1 | -528.99 | 91.90 | -68.20 | 3.60 | -146.94 | 25.53 | -18.94 |
| DREAM | 137.6 | 30 | 2593 | 2617 | 2623 | 2646 | 5269 | 0.01 | 3 | -2094.92 | 192.10 | -162.33 | 3.60 | -1745.76 | 160.08 | -135.27 |
| PHARAO | 134.4 | 6 | 499 | 497 | 505 | 503 | 1008 | 0.01 | 3 | 21167.92 | 74.41 | -74.94 | 3.60 | 17639.93 | 62.01 | -62.45 |
| MRC_mild_Diuretic | 161.5 | 18 | 4279 | 8545 | 4297 | 8654 | 12951 | 0.01 | 5.5 | 118.96 | 87.35 | 186.39 | 3.60 | 181.74 | 133.46 | 284.77 |
| MRC_mild_β-blocker | 161.5 | 42 | 4361 | 8545 | 4403 | 8654 | 13057 | 0.01 | 5.5 | 327.18 | 147.79 | -1529.95 | 3.60 | 499.86 | 225.79 | -2337.42 |
| DEMAND_ACEI | 147.1 | 0 | 127 | 125 | 127 | 127 | 254 | 0.02 | 3.8 | 63.50 | 26.74 | -169.35 | 3.60 | 67.03 | 28.22 | -178.75 |
| DEMAND_CCB+ACEI | 147.8 | 0 | 126 | 125 | 126 | 127 | 253 | 0.02 | 3.8 | 63.50 | 26.74 | -169.35 | 3.60 | 67.03 | 28.22 | -178.75 |
| AIPRI | 143 | 4 | 296 | 278 | 300 | 283 | 583 | 0.02 | 3 | 230.71 | 40.92 | -63.42 | 3.60 | 192.26 | 34.10 | -52.85 |
| PREVENDIT | 130 | 17 | 414 | 419 | 431 | 433 | 864 | 0.03 | 3.83 | -140.64 | 56.52 | -31.33 | 3.60 | -149.62 | 60.13 | -33.33 |
| BCAPS | 138.9 | 5 | 391 | 384 | 396 | 397 | 793 | 0.03 | 2.99 | 49.70 | 24.51 | -1801.62 | 3.60 | 41.28 | 20.36 | -1496.35 |
| Oslo | 155.75 | 14 | 392 | 366 | 406 | 379 | 785 | 0.03 | 5.5 | -5495.50 | 39.48 | -38.92 | 3.60 | -5495.50 | 39.48 | -38.92 |
| IMAGINE | 121.5 | 45 | 1235 | 1228 | 1280 | 1273 | 2553 | 0.04 | 2.95 | 5172.83 | 68.96 | -70.85 | 3.60 | 4238.84 | 56.51 | -58.06 |
| ADVANCE | 145 | 211 | 5358 | 5314 | 5569 | 5571 | 11140 | 0.05 | 4.3 | 121.31 | 63.72 | 1258.94 | 3.60 | 144.90 | 76.12 | 1503.73 |
| Wolff | 176.7 | 2 | 43 | 40 | 45 | 42 | 87 | 0.05 | 2 | 315.00 | 10.95 | -11.77 | 3.60 | 175.00 | 6.08 | -6.54 |
| CAMELOT_ACEI | 129 | 24 | 649 | 622 | 673 | 655 | 1328 | 0.05 | 2 | 67.93 | 27.35 | -140.50 | 3.60 | 37.74 | 15.20 | -78.05 |
| CAMELOT_CCB | 129 | 25 | 638 | 622 | 663 | 655 | 1318 | 0.05 | 2 | 78.90 | 28.71 | -105.48 | 3.60 | 43.83 | 15.95 | -58.60 |
| QUIET | 123 | 48 | 830 | 819 | 878 | 872 | 1750 | 0.06 | 2.25 | 163.66 | 35.76 | -63.52 | 3.60 | 102.29 | 22.35 | -39.70 |
| STONE | 169 | 29 | 809 | 776 | 838 | 828 | 1666 | 0.06 | 2.5 | 35.47 | 20.47 | 132.44 | 3.60 | 24.63 | 14.22 | 91.97 |
| TOMHS | 140.4 | 30 | 638 | 218 | 668 | 234 | 902 | 0.07 | 4.4 | 42.62 | 16.83 | -80.10 | 3.60 | 52.09 | 20.57 | -97.90 |
| PREVENT | 129.4 | 24 | 393 | 380 | 417 | 408 | 825 | 0.07 | 3.00 | 90.31 | 22.59 | -45.22 | 3.60 | 75.25 | ∞ | -37.68 |
| FEVER | 154.3 | 250 | 4591 | 4518 | 4841 | 4870 | 9711 | 0.07 | 3.33 | 48.46 | 33.09 | 90.43 | 3.60 | 44.82 | 30.61 | 83.65 |
| ORIENT | 141.3 | 21 | 261 | 263 | 282 | 284 | 566 | 0.07 | 3.2 | -1906.86 | 23.44 | -22.88 | 3.60 | -1694.98 | 20.84 | -20.34 |
| HUNAN | 160.5 | 37 | 1003 | 961 | 1040 | 1040 | 2080 | 0.08 | 4.72 | 24.76 | 16.66 | 48.22 | 3.60 | 32.47 | 21.84 | 63.22 |
| NAVIGATOR | 139.7 | 371 | 4260 | 4287 | 4631 | 4675 | 9306 | 0.08 | 5 | 346.94 | 71.41 | -121.38 | 3.60 | 481.86 | 99.18 | -168.59 |
| Syst-Eur | 173.85 | 139 | 2259 | 2098 | 2398 | 2297 | 4695 | 0.09 | 2 | 34.88 | 22.99 | 72.24 | 3.60 | 19.38 | 12.77 | 40.13 |
| VA-II | 163.6 | 8 | 178 | 175 | 186 | 194 | 380 | 0.10 | 3.25 | 18.21 | 9.44 | 253.71 | 3.60 | 16.44 | 8.52 | 229.05 |
| HYVET | 173 | 159 | 1774 | 1710 | 1933 | 1912 | 3845 | 0.11 | 2.10 | 42.75 | 23.91 | 201.72 | 3.60 | 24.94 | 13.95 | 117.67 |
| STOP | 195 | 57 | 755 | 728 | 812 | 815 | 1627 | 0.11 | 2.08 | 27.36 | 15.60 | 110.93 | 3.60 | 15.81 | 9.02 | 64.09 |
| PEACE | 133 | 439 | 3719 | 3668 | 4158 | 4132 | 8290 | 0.11 | 4.8 | 148.93 | 49.68 | -149.28 | 3.60 | 198.57 | 66.24 | -199.04 |
| SHEP-P | 172 | 33 | 410 | 95 | 443 | 108 | 551 | 0.12 | 2.83 | 21.80 | 8.93 | -49.55 | 3.60 | 17.13 | 7.02 | -38.95 |
| Kuramoto | 168.7 | 3 | 35 | 36 | 38 | 41 | 79 | 0.12 | 4 | 23.25 | 5.72 | -11.26 | 3.60 | 25.84 | 6.35 | -12.51 |
| EUROPA | 137 | 633 | 5477 | 5339 | 6110 | 6108 | 12218 | 0.13 | 4.2 | 44.84 | 29.77 | 90.88 | 3.60 | 52.32 | 34.73 | 106.03 |
| Dutch TIA | 157.5 | 93 | 639 | 646 | 732 | 741 | 1473 | 0.13 | 2.60 | 865.09 | 28.38 | -30.37 | 3.60 | 624.79 | 20.50 | -21.94 |
| PROGRESS_ACEI | 144 | 157 | 1124 | 1115 | 1281 | 1280 | 2561 | 0.13 | 3.9 | 157.59 | 31.22 | -51.72 | 3.60 | 170.72 | 33.83 | -56.03 |
| PART | 133 | 33 | 275 | 268 | 308 | 309 | 617 | 0.13 | 4.7 | 39.15 | 13.03 | -38.94 | 3.60 | 51.11 | 17.01 | -50.84 |
| PRoFESS | 144.1 | 1271 | 8875 | 8820 | 10146 | 10186 | 20332 | 0.13 | 2.50 | 113.19 | 55.34 | -2497.73 | 3.60 | 78.61 | 38.43 | -1734.54 |
| SCOPE | 166.25 | 304 | 2173 | 2130 | 2477 | 2460 | 4937 | 0.13 | 3.7 | 87.59 | 33.24 | -137.98 | 3.60 | 90.02 | 34.17 | -141.81 |
| SHEP | 170.3 | 236 | 2129 | 2036 | 2365 | 2371 | 4736 | 0.14 | 4.5 | 24.10 | 16.66 | 43.49 | 3.60 | 30.12 | 20.83 | 54.36 |
| MRC-old_Diuretic | 184.5 | 111 | 970 | 1899 | 1081 | 2213 | 3294 | 0.14 | 5.8 | 25.51 | 16.02 | 62.52 | 3.60 | 41.09 | 25.81 | 100.73 |
| MRC-old_β-bloker | 184.5 | 151 | 951 | 1899 | 1102 | 2213 | 3315 | 0.14 | 5.8 | 205.54 | 33.52 | -49.74 | 3.60 | 331.15 | 54.00 | -80.13 |
| PROGRESS_combination | 149 | 150 | 1620 | 1519 | 1770 | 1774 | 3544 | 0.14 | 3.9 | 16.95 | 12.52 | 26.22 | 3.60 | 18.36 | 13.57 | 28.40 |
| DIABHYCAR | 145.45 | 358 | 2085 | 2100 | 2443 | 2469 | 4912 | 0.15 | 4 | 343.40 | 43.91 | -59.00 | 3.60 | 381.55 | 48.79 | -65.56 |
| ACTION | 137.5 | 580 | 3245 | 3259 | 3825 | 3840 | 7665 | 0.15 | 4.9 | -3012.92 | 63.61 | -61.04 | 3.60 | -4100.92 | 86.58 | -83.08 |
| GISSI-AF | 138.6 | 118 | 604 | 598 | 722 | 720 | 1442 | 0.17 | 1 | 166.40 | 22.49 | -30.83 | 3.60 | 46.22 | 6.25 | -8.56 |
| TRANSCEND | 141 | 455 | 2499 | 2466 | 2954 | 2972 | 5926 | 0.17 | 4.67 | 61.62 | 28.58 | -394.32 | 3.60 | 79.94 | 37.07 | -511.53 |
| HEP | 196.15 | 58 | 361 | 371 | 419 | 465 | 884 | 0.20 | 4.4 | 15.69 | 8.85 | 69.10 | 3.60 | 19.18 | 10.82 | 84.45 |
| HSCSG | 167 | 41 | 185 | 180 | 226 | 226 | 452 | 0.20 | 2.75 | 45.20 | 10.55 | -19.79 | 3.60 | 34.53 | 8.06 | -15.11 |
| I-PRESERVE | 136.5 | 439 | 1628 | 1626 | 2067 | 2061 | 4128 | 0.21 | 4.67 | -756.14 | 42.37 | -38.10 | 3.60 | -980.88 | 54.96 | -49.42 |
| HOPES | 139 | 897 | 3748 | 3479 | 4645 | 4652 | 9297 | 0.25 | 5 | 16.94 | 13.17 | 23.71 | 3.60 | 23.53 | 18.30 | 32.94 |
| EWPHE | 182.5 | 74 | 342 | 315 | 416 | 424 | 840 | 0.26 | 4.7 | 12.63 | 7.42 | 42.22 | 3.60 | 16.49 | 9.69 | 55.12 |
| ACTIVE | 138.3 | 1188 | 3330 | 3306 | 4518 | 4498 | 9016 | 0.27 | 4.10 | 485.80 | 49.37 | -61.96 | 3.60 | 553.27 | 56.23 | -70.57 |
| MICRO-HOPE | 142 | 373 | 1435 | 1260 | 1808 | 1769 | 3577 | 0.29 | 4.5 | 12.28 | 9.13 | 18.77 | 3.60 | 15.35 | 11.41 | 23.47 |
| IDNT-CCB | 158.5 | 233 | 334 | 347 | 567 | 569 | 1136 | 0.39 | 2.60 | -48.13 | 27.62 | -12.86 | 3.60 | -34.76 | 19.95 | -9.29 |
| IDNT-ARB | 159 | 189 | 390 | 347 | 579 | 569 | 1148 | 0.39 | 2.60 | 15.69 | 8.40 | 119.49 | 3.60 | 11.33 | 6.06 | 86.30 |
| RENAAL | 152.5 | 297.00 | 454.00 | 426.00 | 751.00 | 762.00 | 1513 | 0.44 | 3.4 | 21.99 | 10.51 | -239.01 | 3.60 | 20.77 | 9.93 | -225.73 |

**Abbreviation:** Base_SBP, systolic blood pressure at baseline; a, the number of people who fulfilled the MACEs in treatment group; b, the number of people who did not fulfill the MACEs in treatment group; c, the number of people who fulfilled the MACEs in control group; d, the number of people who did not fulfilled the MACEs in control group; n1, the number of people in treatment group; n2, the number of people in control group; n, the total number of people in the trial; CER, control events rate; Y-S, the ratio of the observed follow-up duration to the mean follow-up duration among the included trials; NNT, the number needed to treat; NNT_CL, the lower 95% confidence limits for the NNT; NNT_UL, the upper 95% confidence limits for the NNT; NNT_S, the NNT after standardization; NNT_S_CL, the lower 95% confidence limits for the NNT after standardization; NNT_S_UL, the upper 95% confidence limits for the NNT after standardization.

# Figure S3. Results of Sensitive Analysis

**
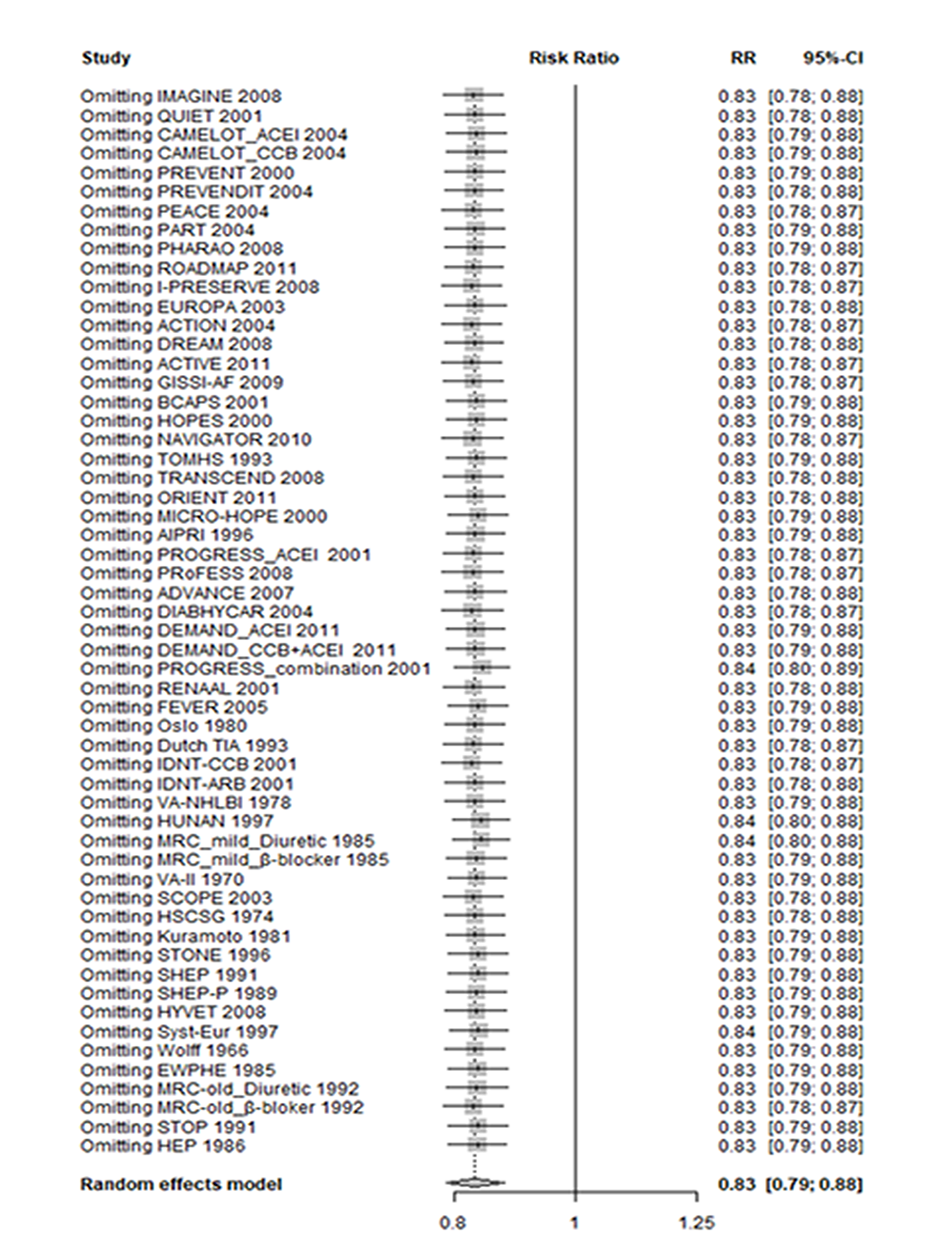
**

# Table S5. Results of meta-regression

| **Potential factors** | **P>\| t \|** | **95% confidence interval** |
| --- | --- | --- |
| Mean age at baseline | 0.217 | (0.9919764, 1.034095) |
| Percentage of female | 0.822 | (0.9923417, 1.009625) |
| Percentage of hypertension | 0.369 | (0.9972013, 1.007275) |
| BMI | 0.032 | (1.00617, 1.1316) |
| Baseline SBP | 0.143 | (0.98566, 1.00224) |
| Baseline DBP | 0.515 | (0.9868115, 1.026056) |
| Follow-up duration | 0.737 | (0.9494623, 1.074838) |

**
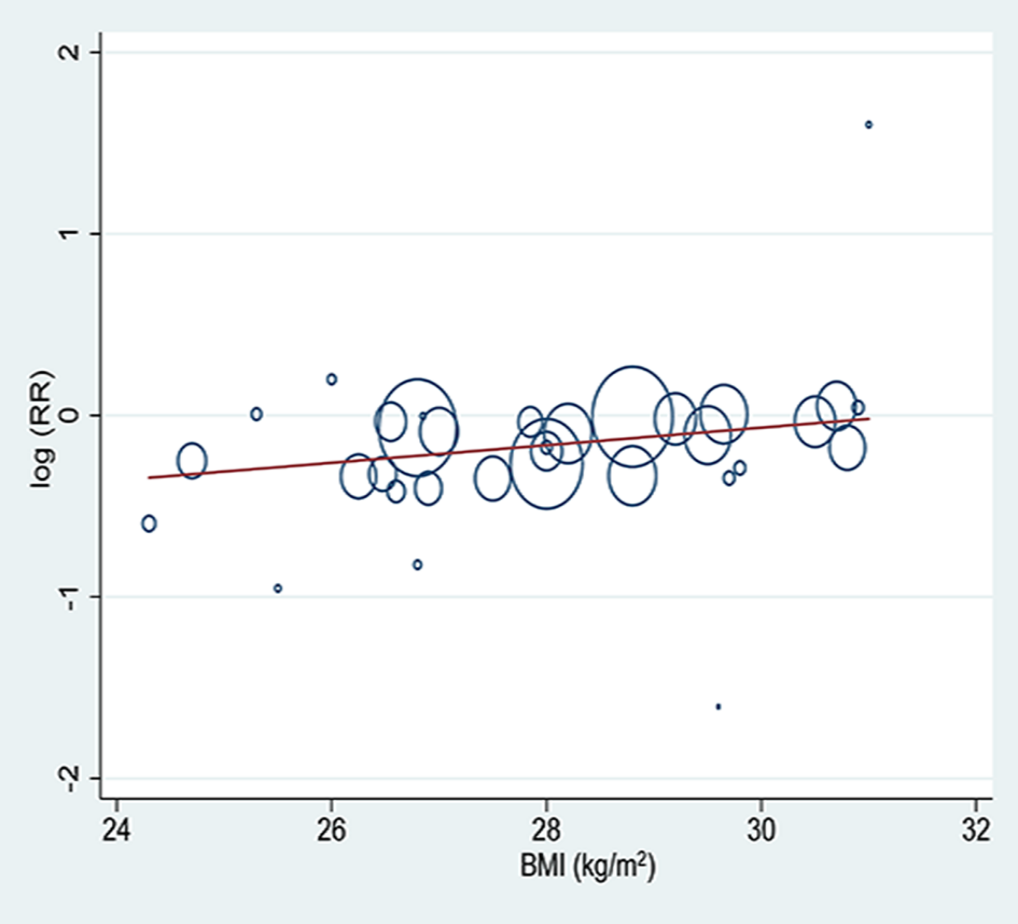
**

**The effect of BMI on the results**

# Figure S4. Results of publication bias

**
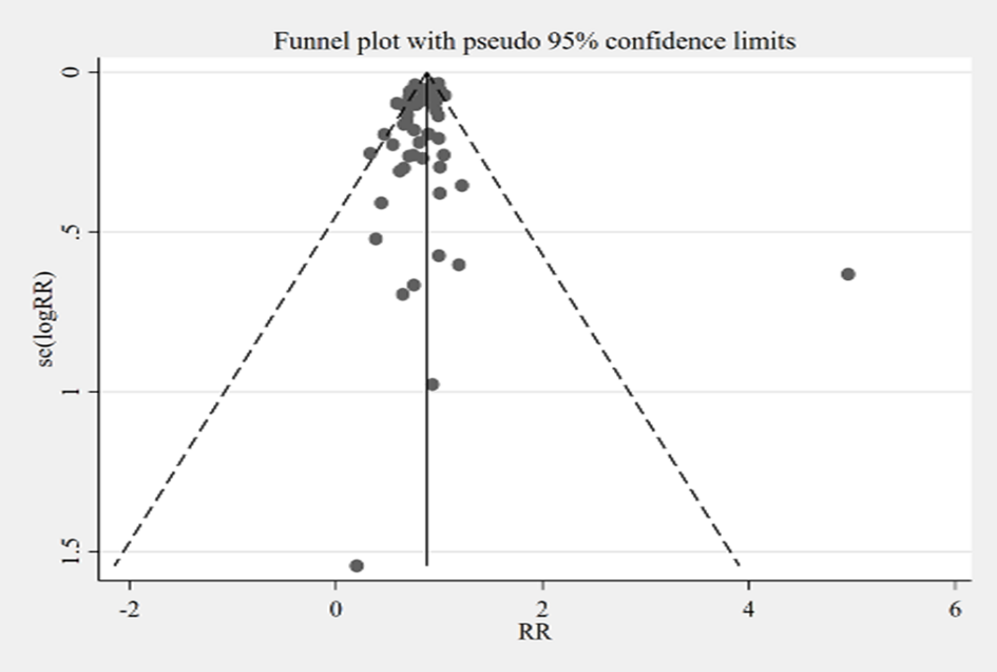
**

**Standard funnel plot**

**
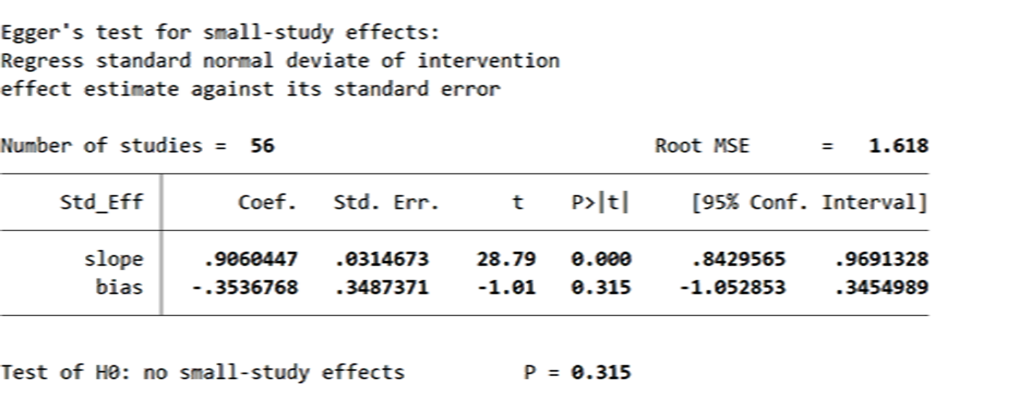
**

**Result of Egger test**
